# Go with the flow: The extent of drag reduction as epiphytic bromeliads reorient in wind

**DOI:** 10.1371/journal.pone.0252790

**Published:** 2021-06-24

**Authors:** Jessica Y. L. Tay, Gerhard Zotz, Jaroslaw Puczylowski, Helena J. R. Einzmann

**Affiliations:** 1 Functional Ecology of Plants, Institute of Biology and Environmental Sciences, University of Oldenburg, Oldenburg, Germany; 2 Smithsonian Tropical Research Institute, Panama, Republic of Panama; 3 ForWind – Center for Wind Energy Research, University of Oldenburg, Oldenburg, Germany; USDA Agricultural Research Service, UNITED STATES

## Abstract

Vascular epiphytes represent almost 10% of all terrestrial plant diversity. Being structurally dependent on trees, epiphytes live at the interface of vegetation and atmosphere, making them susceptible to atmospheric changes. Despite the extensive research on vascular epiphytes, little is known about wind disturbance on these plants. Therefore, this study investigated the wind-epiphyte mechanical interactions by quantifying the drag forces on epiphytic bromeliads when subjected to increasing wind speeds (5–22 m s^-1^) in a wind tunnel. Drag coefficients (*C*_*d*_) and Vogel exponents (*B*) were calculated to quantify the streamlining ability of different bromeliad species. Bromeliads’ reconfiguration occurred first via bending and aligning leaves in the flow direction. Then leaves clustered and reduced the overall plant frontal area. This reconfiguration caused drag forces to increase at a slower rate as wind velocity increased. In the extreme case, drag force was reduced by 50% in a large *Guzmania monostachia* individual at a wind velocity of 22 m s^-1^, compared to a stiff model. This species had one of the smallest *C*_*d*_ (0.58) at the highest wind velocity, and the largest negative mean *B* (-0.98), representing the largest reconfiguration capacity amongst the tested bromeliads. The streamlining ability of bromeliads was mainly restricted by the rigidity of the lower part of the plant where the leaves are already densely clustered. Wind speeds used in this study were generally low as compared to storm force winds. At these low wind speeds, reconfiguration was an effective mechanism for drag reduction in bromeliads. This mechanism is likely to lose its effectiveness at higher wind speeds when continuous vigorous fluttering results in leaf damage and aspects such as root-attachment strength and substrate stability become more relevant. This study is a first step towards an understanding of the mechanical bottleneck in the epiphyte-tree-system under wind stress.

## 1. Introduction

Vascular epiphytes form a major component in many vegetation types, particularly in tropical latitudes, representing globally almost ten percent of terrestrial plant diversity [1]. Epiphytes are, by definition, non-parasitic but are structurally dependent on trees, taking advantage of previously unexploited spaces such as tree crotches and branches in the canopy [2]. They play a key role in the hydrology of many tropical forests [3–5], influence the circulation of nutrient fluxes [6–8] and provide favourable micro-climates and refugia for other biota [9, 10]. Epiphytes are closer to the interface of vegetation and atmosphere than understorey herbaceous plants, potentially making them more susceptible to changes in atmospheric conditions [e.g., 11–13]. Despite the extensive research on the general functional ecology and biology [14–19] of vascular epiphytes, the physical disturbance caused by wind on epiphytic growth has received very little scientific attention. There are two ways in which wind acts as a mechanical disturbance to epiphytes—either by directly dislodging individuals from their substrate or by breaking the substrate itself. In both cases, survival on the ground is transitory and premature death is almost inevitable [20].

Wind profiles under non-storm conditions are available for a few tropical forests. For example, in the lowland forests of Northern Colombia and West Malaysia, mean wind speeds within the forest range from < 1 to ca. 5% of the mean wind speed measured above the forest (i.e. 3.5 m s^-1^ at 60 meters above the ground in Colombia [21] and 2.8 m s^-1^ at 47 meters above the ground in Malaysia [22]). Not surprisingly, wind speeds increase along the vertical gradient from the ground to the canopy. While it is very unlikely that the mentioned wind speeds remove an individual epiphyte or break a branch, a similar conclusion cannot be drawn in the event of a tropical storm with higher wind speeds, e.g., gale force winds of 17.2–24.4 m s^-1^ and storm force winds of 24.5–32.7 m s^-1^, according to the Beaufort scale [23]. For storms with wind speed > 33 m s^–1^, classified as hurricanes or typhoons (depending on the region), forest damage is often extensive [**S1 Table in** S1 File].

Currently, no study specifically quantifies the direct effects of wind on vascular epiphytes. Our understanding of the impact of storms on epiphyte community structure and dynamics is still limited. A comprehensive literature search yielded about 25 reports on storm impacts on epiphytes [**S2 Table in** S1 File], with the majority of them focusing on hurricanes or ‘storms’ with unspecified strength and duration [24–26]. It is certainly interesting to examine how ‘strong winds’ influence epiphyte assemblages. However, storms of such intensity are rare and epiphytes experience lower wind speeds during most of their lifetime. That said, wind directions below the canopy can be very erratic [21] and wind flow in the upper canopy and directly above forests is highly turbulent. Therefore, forces acting on the plants in the forest are not simply a function of the mean wind speed, and forces exerted by wind gusts can be ten times greater than those due to mean wind speed [e.g. 24, 27, 28]. The impact of such wind disturbances can be exacerbated due to ongoing forest fragmentation that results in abrupt and more open forest edges [29]. In such forest stands wind gusts penetrate deeper as compared to dense forests with naturally grown edges [30]. Furthermore, global climate change is expected to intensify the magnitude of natural tropical storms [31, 32]. With these factors, previously sheltered epiphytes would be more exposed to higher wind loading, making it more important than before to understand the effect of wind on these plants.

To assess if wind is a potential threat to epiphytes, the force required to dislodge the epiphytes or to break the substrate must be known. Both are difficult questions to answer since every natural system is complex and wind regimes differ between forest types. Nonetheless, in order to understand the ecological adaptation of epiphytes to their environment, it is essential to study the fundamentals of the wind-epiphyte mechanical interactions, before further investigating if wind could be a potential threat. Thus, as a first step, we use a lab-based approach to study the dynamics of epiphytes in wind, assessing the drag forces these plants experience when subjected to wind ranging from moderate breeze (5–8 m s^-1^) to gale-like winds (17–22 m s^-1^) [23].

### 1.1 Drag force from mechanical loading by wind

The effect of wind on plants has garnered a considerable body of literature over the years as reviewed by de Langre [33], ranging from the plant physical behaviour under wind loading to the effects of wind on plant growth and function. Winds or currents in a flow-dominated habitat are a major stress component for sessile organisms in general [34, 35]. To adapt to external mechanical stress, plants respond with either a growth reaction or a rapid response such as structural reconfiguration [36]. In highly dynamic habitats exposed to intermittent and high flow velocities, an adaptive mechanism by growth reaction is often too slow to reduce the mechanical loading. In such cases, fast adjustments from structural reconfigurations in response to short and sudden events like wind gust or large waves [37, 38] are necessary to reduce the resultant drag force effectively. Moreover, plants under mechanical loading typically have some degree of plasticity, i.e., they deform over time due to exposure to mechanical stress; and elasticity, i.e., they revert to their original form after removal of the applied force.

The drag force from wind, at air temperature of 20°C, *F*_*d*_ (Newton = kg m s^-2^), on a single rigid drag-producing body (hereafter simply referred to as drag body) is defined by Eq 1:

$$F_{d}=\frac{1}{2}\rho A_{c}C_{d}v^{2}.$$ (1)

where *ρ*: air density (kg m^–3^), *A*_*c*_: reference area of the drag-producing body (m^2^), *C*_*d*_: the dimensionless drag coefficient that quantifies the drag on an object from wind, *v*: wind velocity (m s^–1^). A lower *C*_*d*_ value indicates a smaller aerodynamic drag. Numerous tests on isolated standard drag bodies, i.e., spheres, cylinders, disks, have established the relationship of *C*_*d*_ with flow [39–41]. At Reynolds numbers from 1 x 10^3^–3 x 10^5^, *C*_*d*_ can be regarded as a constant. In this case, a quadratic relationship is observed between drag force and velocity [42] where:

$$F_{d}\approx \;v^{2}$$ (2)

As *C*_*d*_ is a quantity that depends on the shape of an object, for flexible bodies such as typical plants, *C*_*d*_ is commonly not constant over larger spans of Reynolds numbers [33, 43]. At higher aerodynamic loading, most plants undergo some form of reconfiguration, reducing the frontal area and achieving a more streamlined shape. This changes *C*_*d*_ subsequently and lowers the overall *F*_*d*_ on themselves [37, 44, 45]. For example, broad leaves reconfigure into cones with increasing wind speeds [38]; in aquatic systems, macrophytic freshwater plants go from an upright to a horizontal position close to the substratum at increasing flow rates [46]. Through the process of reconfiguration, numerous studies found that drag load increases more slowly over increasing flow velocities. Thus, the measured force-velocity relationship for vegetation deviates from the quadratic relationship [e.g., 47–49]. The extent to which the force-velocity relationship deviates from the second-power relation can be described by the Vogel exponent *B* [37, 50], which modifies the above Eq 2 as follows:

$$F_{d}\approx \;v^{2+B},$$ (3)

The more negative the Vogel exponent, the larger the extent of drag force reduction with increasing velocity. Therefore, it is used to quantify the effect of reconfiguration of the drag body for streamlining [34, 51]. Vogel [38] found that structural reconfiguration caused drag to increase more or less linearly with wind flow (i.e., *B* ~ −1). For example, wind/water channels or on-site wind and currents were used on different types of vegetation such as trees, flowers and submerged macrophytes to determine the respective Vogel exponent. Entire poplar trees with leaves had a *B* value of -0.71 [52]; that of terrestrial herbaceous plants such as the daffodil and fountain grass was -0.60 and -0.52, respectively [48]; and brown seaweed in the wave-swept intertidal had values of -0.93 to -0.86 [51].

This study is the first to investigate experimentally the mechanical interactions of wind and epiphytes. Specifically, we assessed the effect of wind on five species of bromeliads by quantifying the resulting drag forces on these plants. We expected that bromeliads would undergo some reconfiguration, hence causing drag to increase at a slower rate as wind velocity increased. This paper highlights two main aspects: 1) the relationship between *C*_*d*_ of bromeliads and increasing wind velocity and 2) the Vogel exponent as a measure of the effect of streamlining that reduces the extent of drag force increment over increasing wind loading. By doing so, the influence of wind and bromeliad morphology on the extent of reconfiguration will be explored. This paper concludes with a discussion on the implication of drag loading as a mechanical disturbance on epiphytes in nature and highlights some caveats of the study.

## 2. Materials and methods

### 2.1 Preliminary testing of setup and equipment

**Fig 1:**
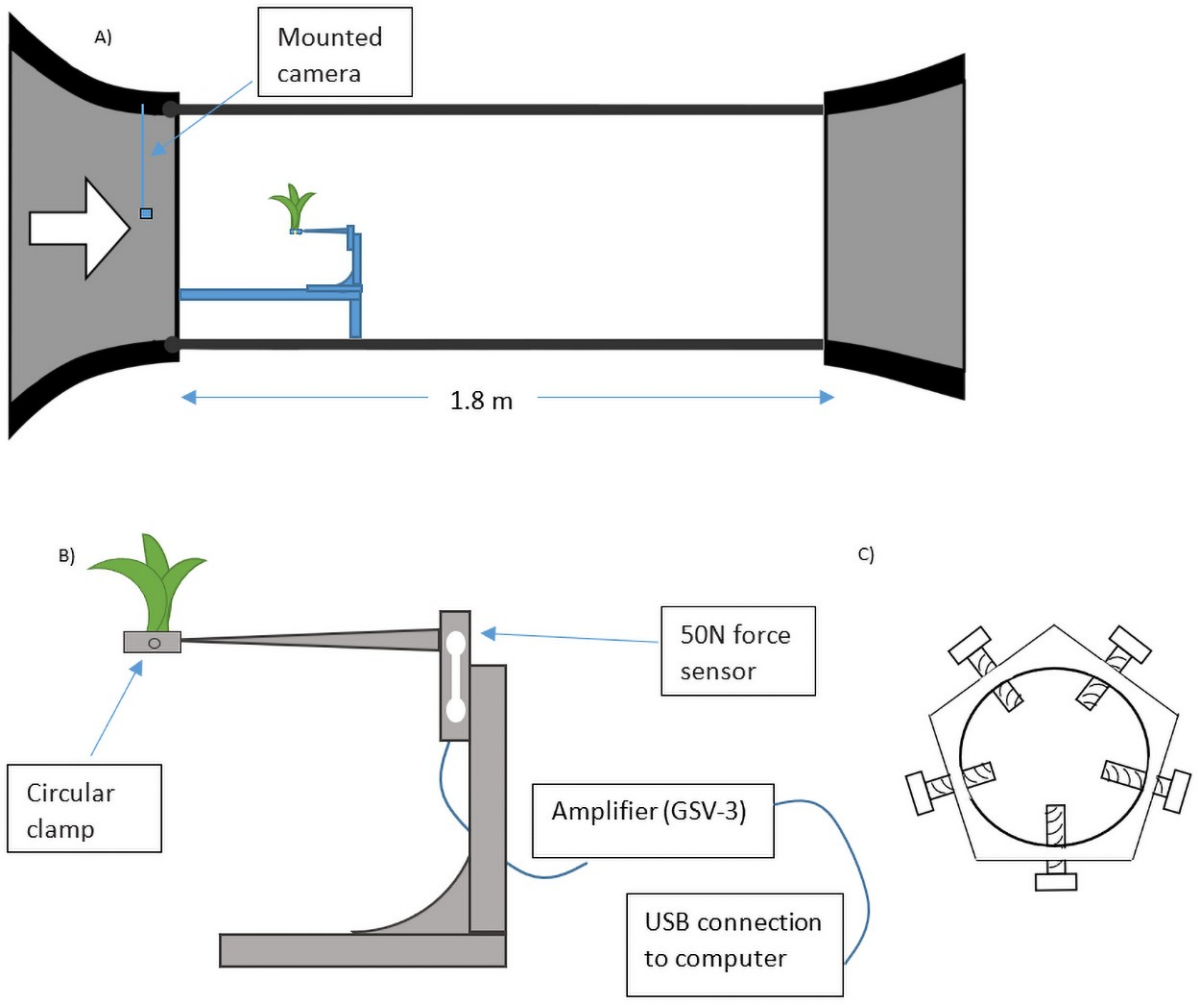
Wind tunnel setup and equipment used for drag force testing. (A) Wind tunnel setup. Each drag body is positioned in the middle of the wind tunnel. A small camera was mounted upstream to take frontal photographs of the drag body. (B) Diagram of setup used for testing drag force. Each plant was held in place with a circular clamp, and a scale bar was placed next to the screw on the clamp for measuring the size of the plant. Drag force was recorded with a 50 N double bending beam force sensor that was connected to a computer via an amplifier. (C) Diagram of the clamp (Ø = 50 mm, Ø = 9 mm when all screws are fully screwed in) used to mount plant on the setup where screws are adjustable to fit different plant sizes.

The experimental data of this paper were all obtained from wind tunnel drag force tests in the physics lab, TWiSt, within the ForWind research centre at the University of Oldenburg. During the experiments, each drag body was positioned in the middle of a wind tunnel, which was used in a free stream configuration (Fig 1A). A circular 5-screws clamp was used to hold the drag body in place (Fig 1C). The wind tunnel is an open loop and the nozzle outlet had a cross section of 0.8 x 1 m^2^ and a test section of 1.8 m. The wind tunnel generates free stream velocities of up to 22 m s^-1^ that was determined by means of prechamber pressure measurements using a pitot tube. The turbulence intensity of the wind tunnel is 0.2%. Since the test object is always only 10 cm from the nozzle outlet, the flow around the test object can be considered as laminar flow. To measure the drag force on the drag body, a 50 N double bending beam force sensor (KD140-50N, nominal force ± 50 N, accuracy 0.1%, ME-Meßsysteme, Hennigsdorf, Germany) was connected to a computer via an amplifier (GSV-3), and readings were assessed with the corresponding GSVmulti software (ME-Meßsysteme, Hennigsdorf, Germany, Version 1.43) for live viewing and recording of data (Fig 1B).

The aerodynamic characteristics of five smooth spheres with a diameter range from 8–20 cm were documented, to verify the applicability of the wind tunnel setup used in this study. Given the range of wind velocities and sphere sizes used, *C*_*d*_ values in the flow regimes before the drag crisis occurred were of interest for this study. The Reynolds number (Re) was calculated as:

$$Re=\frac{vL}{\gamma }$$

where *v*: wind velocity (m s^–1^), L: characteristic linear dimension of the object, and in this case, it is the length (m) of the drag body (i.e., diameter of the sphere) and *γ*: kinematic viscosity of air. Based on the sphere diameters and that the measurement was taken at 20°C, the Re range is between 6 x 10^3^ and about 2.9 x 10^5^ and the *C*_*d*_ value is reported to be around 0.45–0.50 [39, 53, 54]. Using the wind tunnel setup described above, steady sphere drag was measured through the wind velocity range of 1 to 22 m s^-1^. At each wind speed, drag force was averaged over 30 seconds (sampling rate of 50 times s^-1^). Since the accuracy of the load cell is at 0.1% (i.e., 0.05 N), drag forces measured at very low wind speeds from 1–4 m s^-1^ were < 0.05 N. Therefore, drag force measurements taken at those low wind speeds were omitted from all further analyses due to measurement inaccuracies. The *C*_*d*_ values of the spheres were calculated using Eq 1. The *C*_*d*_ values of the spheres were around 0.43–0.54 [**S1 Fig in** S1 File], close to the above-mentioned reference *C*_*d*_ values, with the exception of the 8 cm sphere. Since *C*_*d*_ is shape dependent, once the diameter of the sphere gets too small relative to the size of the support pole behind it, the dominant shape is no longer spherical. Instead, the influence of the cylindrical shape of the support pole, which itself had a higher drag coefficient of about 0.8, increases. This served as an indication that force data measured for epiphytes with a length and width ≤ 8 cm should be interpreted with caution because the setup might not be able to document the correct drag forces of such small plants. Acknowledging this caveat, the wind tunnel setup generated reliable experimental data and is suitable for further investigation with the epiphytes.

### 2.2 Wind tunnel measurements with epiphytes in the University of Oldenburg

#### 2.2.1 Ethics statement

Permission to work in the Barro Colorado Nature Monument was granted by the Smithsonian. Tropical Research Institute. A permit to export plants was granted by the Panamanian authorities (SEX/P-3-19).

**Fig 2:**
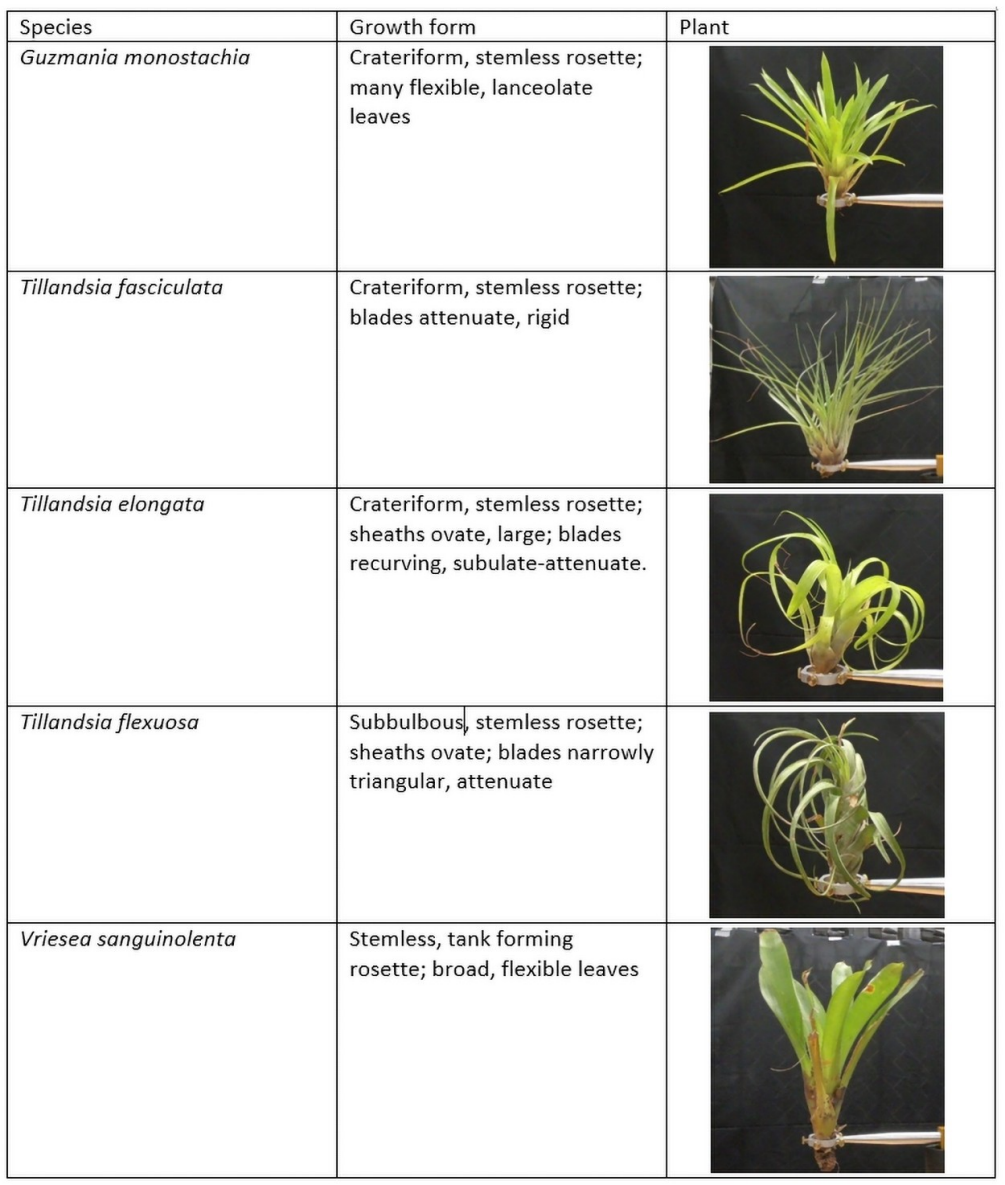
Growth form description of the studied bromeliads.

We collected 112 individuals of five epiphytic bromeliads of varying size. Those five species represent varying growth forms (Fig 2). *Tillandsia flexuosa* (n = 24) was collected from trees in public areas in the Veraguas province in Panama. The other four species (*Guzmania monostachia* (n = 24), *Tillandsia fasciculata* (n = 24), *Vriesea sanguinolenta* (n = 20), *Tillandsia elongata* (n = 20)) were collected from *Annona glabra* trees on Lake Gatun in the areas around Barro Colorado Natural Monument, Panama. All plant names follow the Plant List [55]. Bromeliads were chosen for this study because they have a relatively predictable clear form when subjected as a whole plant to oncoming wind. In contrast, epiphytes that are growing very close to their host will not provide much of a barrier against the wind and thus measuring them individually without their host is rather irrelevant. Before export to Germany, plants were carefully cleaned and rinsed to remove all organic material trapped in the leaf axils. In Oldenburg, Germany, the plants were kept in the greenhouse under moist tropical conditions when not used in experiments. Wind tunnel measurements were completed within a week after arrival from Panama.

Prior to clamping (Fig 1B), all dead leaf parts were removed. Roots were carefully trimmed in order to ensure that measurement of drag force was only on the leaves, as these parts are more prominently exposed to wind flow in nature. Drag force measurements were done with the same force sensor and computer-amplifier setup as the trial tests with spheres. Before each measurement, the longest leaf (LL) of each individual plant was recorded and the force sensor tared. None of the plants tested had a length and width ≤ 8 cm and, therefore, the dominant shape for determining *C*_*d*_ should not be affected. Force measurements were recorded by exposing the plants to wind velocities from 5 to 22 m s^-1^, with stepwise increments of 1 m s^-1^. Plants were exposed to each wind speed for 30 seconds. Wind velocity stabilised after 10 seconds and mean drag force was recorded over the next 20 seconds (sampling rate of 50 times s^-1^). Due to the mounting of the plants, the setup was more exposed than that of the mounting of the spheres. Thus, the drag on the setup alone was measured separately without the plant. These offset values were subsequently subtracted from the total drag of each sample [**S2 Fig in** S1 File].

To determine the plant frontal area exposed to wind, a small industrial camera (camera: DFK 27AUJ003, 10.7 MP, The Imaging Source, Bremen, Germany; lens: T0412FICS, 1/3" 4mm f1.2 Monofocal, Computar, CBC America LLC) was mounted in the upstream section of the wind tunnel to photograph the plant at each wind velocity (Fig 1A). A portable flood light helped to keep plants brightly lit to maximise the colour contrast against the dark background of the wind tunnel. A scale bar of 1.5 cm was placed on the screw of the clamp, which was visible in all photographs, to establish an image scale. The plant frontal area was determined using Fiji, a distribution of ImageJ for scientific image analysis (version 1.53c) [56]. Additional editing to increase contrast between the plant and the background was done in GIMP (version 2.10.10), if deemed necessary. An additional camera installed at the side of the setup took side-view photographs of the bending and reconfiguration of each plant in increasing wind velocities.

### 2.3 Wind tunnel testing with inflexible models

To study the effect of plant flexibility on the drag scaling, a rigid benchmark test was required. For this, we produced artificially stiffened plants. Due to the different growth form of the species, some were innately more flexible than others. For these species (*G*. *monostachia*, *V*. *sanguinolenta* and *T*. *elongata*), two intermediate-sized specimens were selected to obtain data on the rigid benchmark reference to understand the general trend. For the other two species, *T*. *flexuosa* and *T*. *fasciculata*, which were already naturally stiffer, five specimens were selected each, spanning the size range of the unmanipulated specimens. Following the idea of Aberle and Dittrich [57] who created artificial plants with wires, wooden sticks and resin, we stiffened the plants using a multistep process [**S1 Text in** S1 File]. Those stiffened plants were tested with the same procedure in the wind tunnel as the unmanipulated plants.

### 2.4 Data analysis

The *C*_*d*_ of each unmanipulated and stiffened individual was calculated using Eq 1. The relationship of *C*_*d*_ of unmanipulated plants and wind velocity was then assessed with linear regressions of the log-transformed data.

To evaluate the extent of drag reduction from reconfiguration, the stiff artificial models were used to obtain the rigid benchmarked force to investigate if they follow the velocity-squared fit relationship, i.e., Eq 2, as observed in rigid drag bodies. The overall *C*_*d*_ of each stiffened plant was assumed to be the value which plateaued over the higher wind speeds. This *C*_*d*_ constant, together with the constant reference area of the drag body of the plant and air density, was used to calculate the fitted relationship, with velocity as the only variable. If the wind tunnel measurements on the stiffened plant follows the fit well, then the rigid drag forces of other individuals, for which we had no stiffened model, could be computed theoretically using Eq 1, keeping the projected frontal area of the plant constant (obtained from photographs of plants in the wind tunnel). By comparing the measured drag force on the flexible plants with the rigid benchmarked force, drag reduction by flexibility and reconfiguration was quantified.

The Vogel exponent, *B*, was calculated to quantify the extent to which reconfiguration causes the drag-velocity relationship to deviate from the second-power relation, i.e., Eq 3. It was computed as:

$$B=\frac{log(\frac{F_{d}}{v^{2}})}{\text{log}\left(v\right)}$$ (4)

where *B* is defined as the slope of a log-log plot of a velocity-specific drag as a function of velocity [58]. A lower (more negative) exponent indicates a more effective reconfiguration. To evaluate how the effectiveness of reconfiguration changes over increasing wind velocities, a breakpoint analysis was performed for all individuals with piecewise regression to statistically estimate the wind speed at which the slope of the regression changed.

All statistical analyses were done in R (version 3.6.3) [59].

## 3. Results

**Fig 3:**
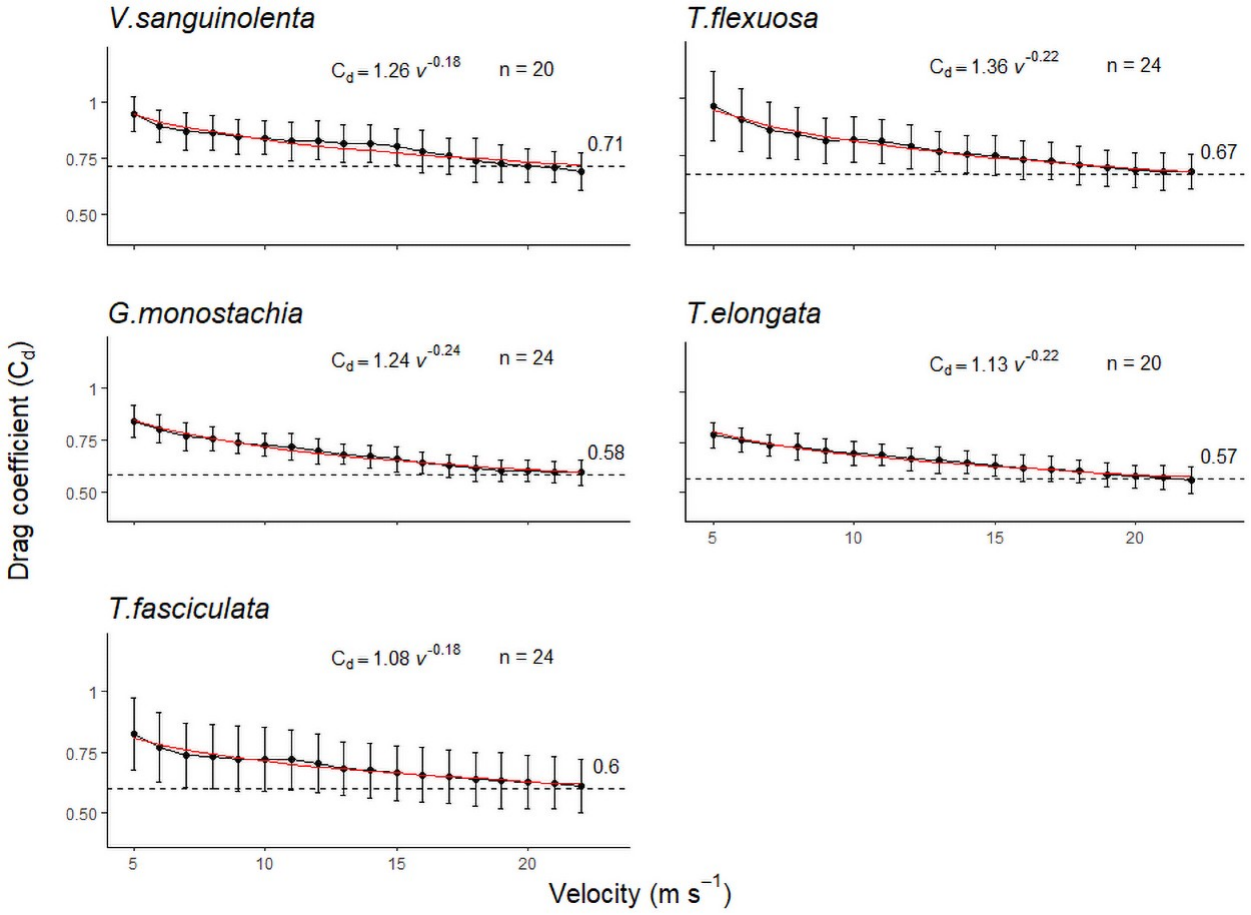
Variations in *C*_*d*_ values over increasing wind speeds. Filled circles are mean velocity-specific C_d_ values of all tested individuals within each species and error bars depict standard deviations. Red lines show the fitted function C_d_(v). Dashed lines indicate the mean C_d_ value of each species, which plateaued over the higher wind speeds at 20–22 m s^-1^. A lower C_d_ indicates a more streamlined shape for less aerodynamic drag.

As wind velocity increased, each individual plant underwent a few stages of reconfiguration by leaf clustering [**S4 Table in** S1 File]. At low velocities, individual leaves began to rotate slightly, resulting in varied angles of attack by wind on the leaves. Hence, at low velocities, larger variations of *C*_*d*_ values were observed (Fig 3). At higher velocities, there was the onset of flutter: leaves started to bend and got reoriented in the direction of flow. As velocity continued to increase, leaves started to cluster and faced downstream [**S4 Table in** S1 File]. During all wind tunnel experiments, plants invariably showed minimal damage on their leaves despite the reconfiguration process. At the end of the measurement, when wind flow was turned off, plants showed elasticity by returning to their original upright orientation.

The drag force (*F*_*d*_) that bromeliads experienced at the maximum wind speed of 22 m s^-1^ permitted by the wind tunnel ranged from < 0.5 N in the smallest juveniles of all species and a maximum of 9 N for a large *T*. *elongata*. The calculated *C*_*d*_ values varied at each wind speed in all five species. The velocity-specific average *C*_*d*_ values (i.e., at each velocity, average *C*_*d*_ from all individuals), were used to determine the fitted function between *C*_*d*_ and velocity, thus obtaining the function *C*_*d*_(*v*), which was species-specific (Fig 3).

The data structure did not allow parametric test, thus an ANCOVA was unsuitable to test for possible interactive effects of species and individual plant size on the *C*_*d*_ values. A non-parametric test showed that within each species, *C*_*d*_ values did not vary with plant size (Kruskal-Wallis, p _(LL~ Cd)_ > 0.1). Between species, *C*_*d*_ values differed significantly at each wind velocity (Kruskal-Wallis, p _(species~ Cd)_ < 0.05). The slope of the relationships of the logarithms of the mean *C*_*d*_ and velocity was invariably < 1 (Pearson Product moment correlation, p < 0.001, Table 1). *Guzmania monostachia* had the steepest slope, indicating that it underwent the greatest reconfiguration with increasing wind velocity. This species had also one of the lower *C*_*d*_ values of around 0.58 (Fig 3). A lower *C*_*d*_ value indicates that the reconfiguration achieved a more streamlined shape reducing aerodynamic drag.

**Table 1:** Linear regression analyses of the relationship between the logarithms of the mean velocity-specific drag coefficient on the bromeliad (*C*_*d*_) and wind velocities (*v*) for five epiphytic bromeliads (y = log(*C*_*d*_); x = log(*v*)).

Species	Linear regression equation	95% confidence interval	Adjusted R^2^	p value
*G*. *monostachia*	y = -0.24x + 0.22	-0.24 ± 0.012	0.99	< 0.001
*V*. *sanguinolenta*	y = -0.19x + 0.24	-0.19 ± 0.024	0.91	< 0.001
*T*. *elongata*	y = -0.22x + 0.14	-0.22 ± 0.015	0.98	< 0.001
*T*. *fasciculata*	y = -0.18x + 0.08	-0.18 ± 0.013	0.97	< 0.001
*T*. *flexuosa*	y = -0.23x + 0.31	-0.23 ± 0.011	0.99	< 0.001

R^2^ and p values are also given.

**Fig 4:**
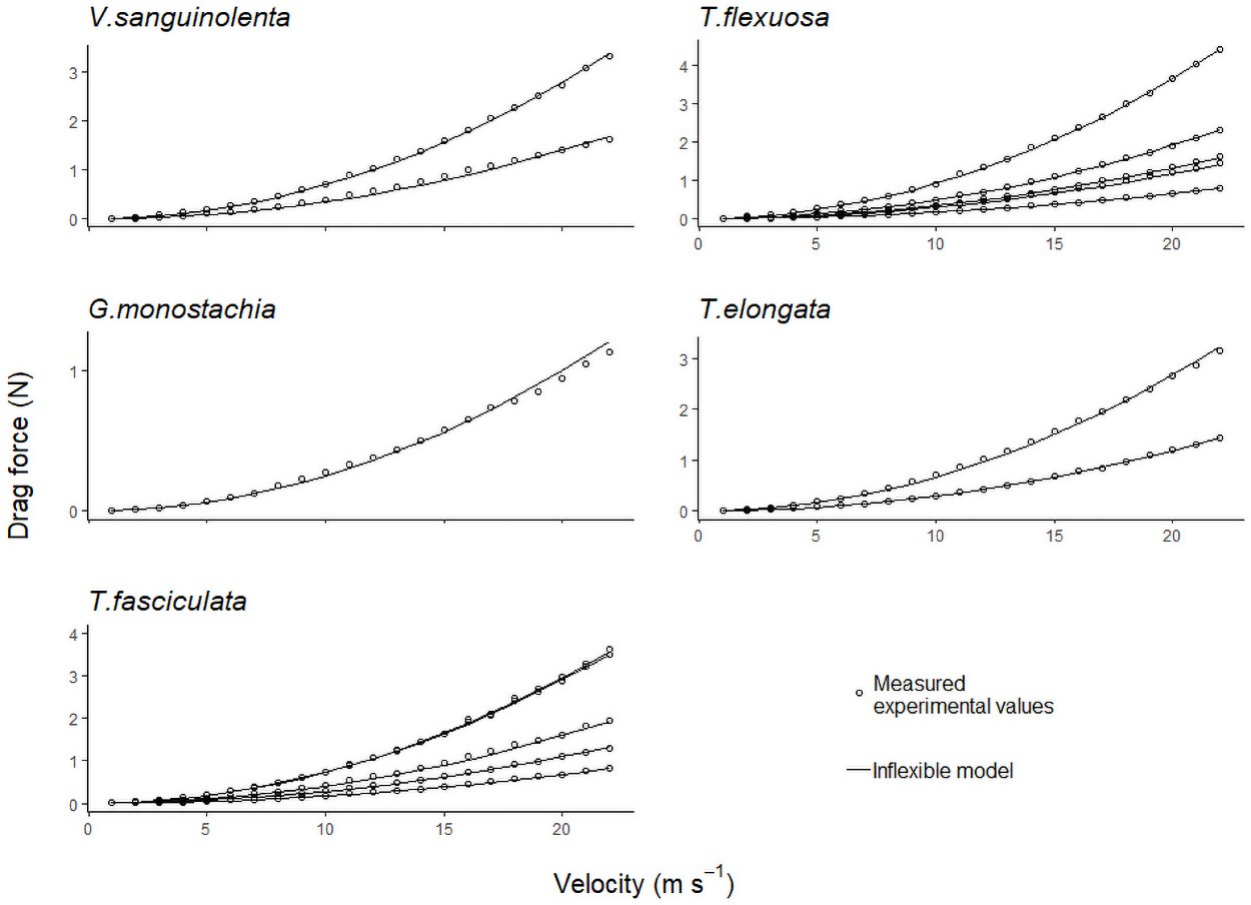
The relationship of drag force and flow velocity. Shown are fitted values (—) of drag force expected on rigid specimens calculated using Eq 1, and measured values (○) on rigid specimens in the wind tunnel.

**Fig 5:**
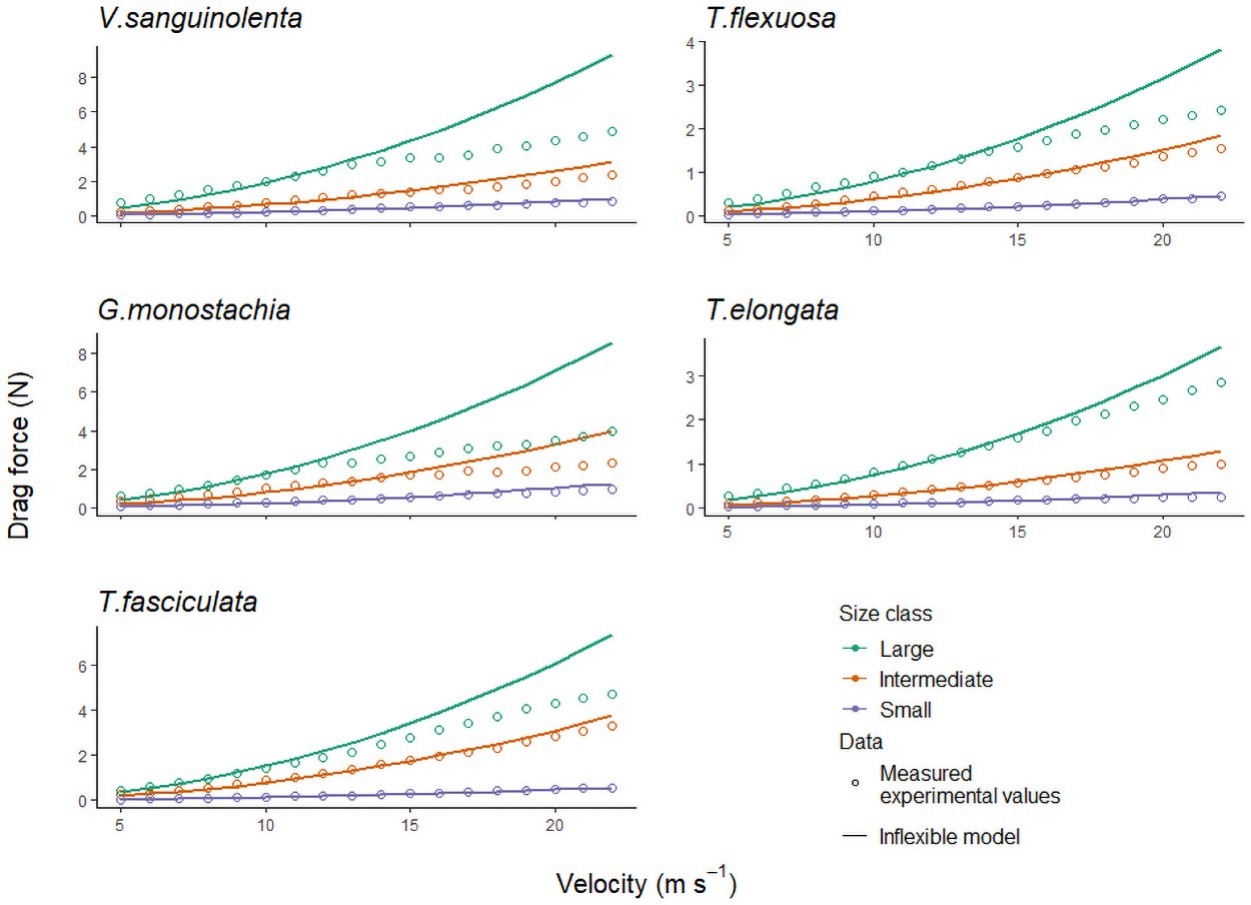
Reduction in drag force on different sized individuals due to reconfiguration. Drag force decreased more in larger individuals than in intermediate and small ones. Small individuals showed little to no reduction in drag forces, when compared to the inflexible models. Large individuals had longest leaf (LL) length ≥ 35 cm; intermediate individuals had 16 cm < LL < 35 cm; small individuals had LL ≤ 16 cm.

The measured drag forces in artificially stiffened plants followed the calculated fitted values well (Fig 4). Therefore, Eq 1 was used to calculate the rigid drag forces of the other plants used in this study. For an inflexible plant, *C*_*d*_ is a constant. This was obtained as the constant value which plateaued over the higher wind speeds (i.e., Fig 3). *C*_*d*_ was obtained for each individual plant and the area of the plant was kept constant using the frontal area of the photograph of each plant at rest (i.e., without wind), assuming that no reconfiguration occurs. The measured drag force on the flexible plants was generally lower than that of the calculated drag force for the rigid plants (Fig 5). Larger individuals (LL > 35 cm) experienced a greater reduction in total drag than intermediate and smaller sized individuals did. For the larger individuals, at wind velocity 22 m s^-1^, drag force was reduced by up to 54% (*G*. *monostachia* (54%), *V*. *sanguinolenta* (47%), *T*. *fasciculata* (36%), *T*. *flexuosa* (37%), and *T*. *elongata* (22%)). For small individuals (LL < 16 cm) however, measured drag force values were mostly close to the rigid drag force (Fig 5). Drag reduction corresponded to frontal area reduction when the plant reconfigured, and hence became more aligned with the flow, i.e., streamlined. Larger individuals generally had a larger overall reduction in frontal area compared to smaller plants [**S3 Table in** S1 File].

**Fig 6:**
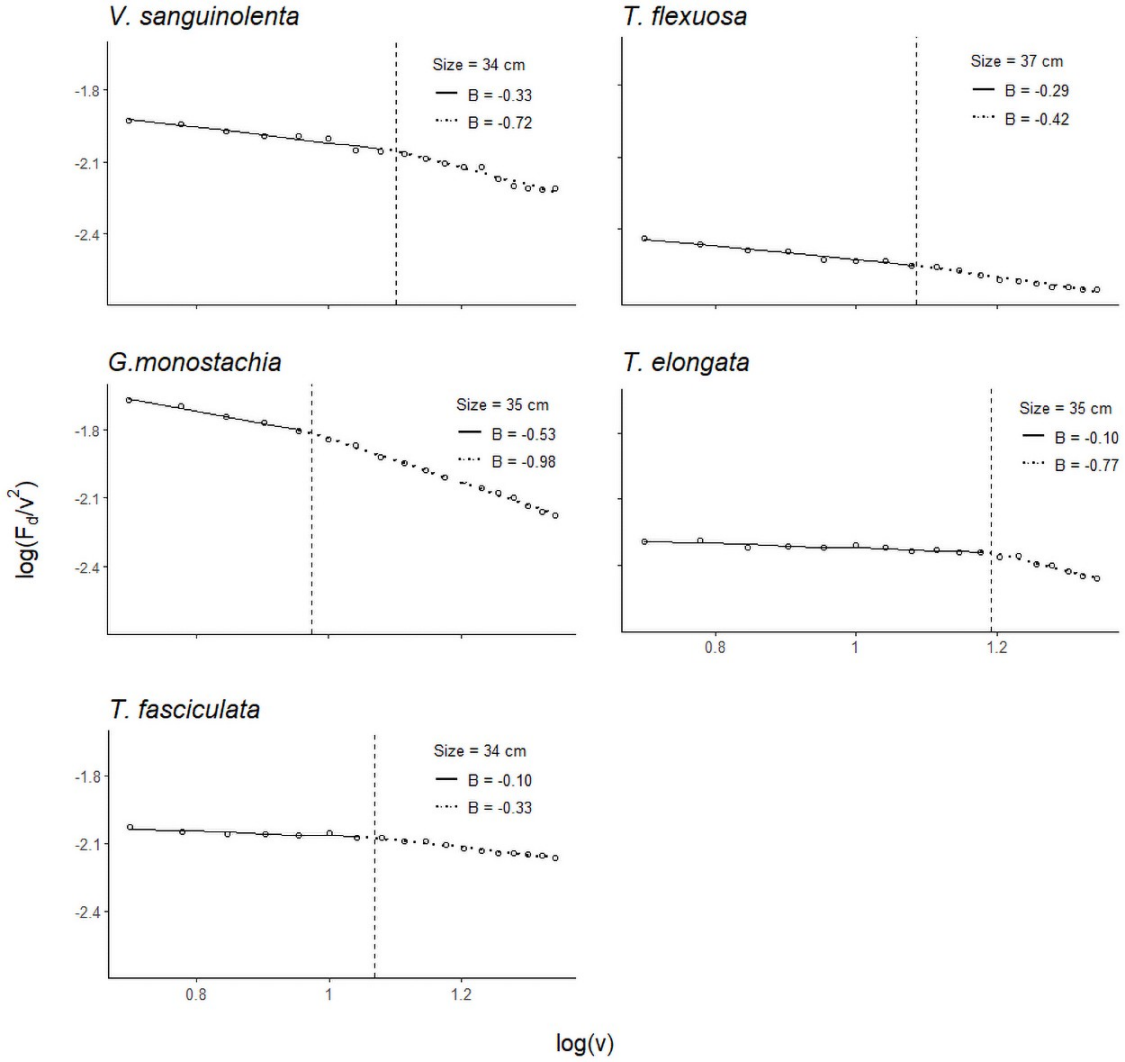
Change in Vogel exponent in a similar sized individual of each species. Dashed vertical lines indicate the breakpoint where the slope of the regression line changes abruptly. This means that at higher wind velocity, drag force is increasing more linearly with wind speed, according to F_d_ ≈ v^2+B^, deviating from the quadratic relationship. Species differ in growth form and flexibility for reconfiguration to reduce drag force, as reflected by the Vogel exponent. Wind speed varied from 5–22 m s.^1^. Regressions for other plant sizes are provided in the **[S3 Fig in** S1 File**]**.

The Vogel exponent was calculated to quantify the extent to which reconfiguration causes the drag-velocity relationship to deviate from the second-power relation, i.e., quantifying the effectiveness of reconfiguration over increasing wind speeds. This can best be described with a breakpoint analysis of the piecewise regression (Fig 6; for all piecewise regressions refer to [**S3 Fig in** S1 File]). *Guzmania monostachia* has the most negative mean Vogel exponents, both before (*B* = -0.45) and after (*B* = -0.98) the breakpoint (Table 2). This indicates that *G*. *monostachia*, among all tested species, reconfigured most effectively. This was also reflected in its low *C*_*d*_ value (Fig 3). The slope of the regression line before the breakpoint was gentler, indicating a small Vogel exponent, i.e., the drag force was still increasing approximately to velocity squared (Fig 6). After the breakpoint, the slope became steeper, indicating a more negative Vogel exponent than that before the breakpoint (Fig 6), suggesting a very pronounced streamlining effect and a nearly linear relationship between drag and velocity. This change in the drag-velocity relationship was observed in all tested individuals. However, the breakpoint analysis did not show a clear pattern as to whether there was a threshold wind velocity before the plant started to be more streamlined (i.e., breakpoint occurred randomly in all individuals of a given species).

**Table 2:** Range of Vogel exponents and size range (LL, longest leaf length) of each species.

Species (n)	Size range (LL, cm)	Range of calculated Vogel exponent	Mean Vogel exponent at slope 1*	Mean Vogel exponent at slope 2
*G*. *monostachia* (n = 24)	11–42	-0.07 –-1.63	-0.45 ± 0.17	-0.98 ± 0.25
*V*. *sanguinolenta* (n = 20)	16–48	-0.08 –-1.92	-0.21 ± 0.23	-0.86 ± 0.42
*T*. *elongata* (n = 20)	16–89	-0.07 –-1.61	-0.23 ± 0.15	-0.83 ± 0.29
*T*. *fasciculata* (n = 24)	9–49	-0.06 –-1.01	-0.20 ± 0.08	-0.32 ± 0.29
*T*. *flexuosa* (n = 24)	11–51	-0.05 –-0.92	-0.30 ± 0.23	-0.38 ± 0.20

*Slope 1 and 2 refer to the regression line before and after the breakpoint, respectively, as indicated in Fig 4.

## 4. Discussion

This is the first experimental study to document the process of reconfiguration for drag force reduction in wind flow by vascular epiphytes. Different species of epiphytic bromeliads, representing different growth forms, were investigated in the wind tunnel to examine how drag forces were reduced over increasing wind velocities. Passive reconfiguration substantially reduced the extent of drag force increment at higher wind velocities on bromeliads compared to inflexible models, in the extreme case up to 54%. Streamlining in the plants was mainly driven by reconfiguration that subsequently reduced the frontal area perpendicular to the wind flow. While measurements, especially in the low wind speeds of 1–4 m s^-1^, could be improved with more sensitive equipment, it would substantially increase costs, but hardly change the fundamental insights in the wind-epiphyte mechanical interactions achieved by this study. The observed trends are more interesting than the exact values themselves.

As wind velocity increased, the leaves of the bromeliads moved in an intricate manner. No actual measurements were taken but we observed (directly during the experiment and from photograph series) that first there was the onset of leaf flutter. As wind speeds further increased, leaves started bending and twisting at irregular intervals. The phenomenon of leaf flutter by torsional galloping is elaborated in [60]. The shape change of the plant body via reconfiguration in turn changes the *C*_*d*_ value [50], thus there is no constant *C*_*d*_ over increasing wind velocity. In this study, the variations in the *C*_*d*_ values of species (Fig 3) reflected the species-specific differences in the ability for passive reconfigurations when subjected to wind. As noted in many studies of wind-plant mechanical interaction, the reconfiguration of a plant causes a reduction in the cross-sectional area that directly reduces total drag load. The bent shape of the plant is typically more streamlined, improving the pressure recovery in its wake thus further reducing drag [33]. This was also observed in this study. *Guzmania monostachia* had the greatest streamlining ability for drag load reduction, having the steepest velocity-related decrease in *C*_*d*_ values, and one of the lowest *C*_*d*_ values at the highest wind velocity (Fig 3; Table 1). Larger plants displayed more pronounced streamlining. In these, the decreased frontal area, when leaves clustered and aligned in the direction of the flow, was the main cause for the reduction in drag force. Smaller plants, with their shorter leaves had little leeway for reconfiguration, hence they did not reduce drag as effectively as larger plants [Fig 5; **S3 Table in** S1 File].

The *C*_*d*_ values for bromeliads tested in this study were around 0.57–0.71 at the highest wind velocity. Most studies on reconfiguration processes with reported *C*_*d*_ values were on trees, marine and freshwater algae and herbaceous plants, e.g., daffodils and grasses [e.g. 45, 51, 61, 62]. We acknowledge that the growth forms and shapes of the test specimens used in those studies with reported *C*_*d*_ values were limited for meaningful direct comparison to epiphytes. Nonetheless, the growth form of the studied epiphytes may be comparable to that of other non-woody plants like daffodils and grasses. It is noteworthy that the *C*_*d*_ values of epiphytes were closer to those of trees. The reported *C*_*d*_ values of daffodils and fountain grass are smaller with 0.40 and 0.30, respectively [62, 63]. This is because of the flexible stem of daffodils that was able to bend at high velocities until it was almost parallel to the wind [62]. In contrast, the rigidity of the bromeliads’ body was the limiting factor against achieving a more streamlined shape. The photograph series [**S4 Table in** S1 File] shows that reconfiguration occurred first via bending and aligning of leaves in the direction of wind flow, and later, at higher wind velocities, clustering of the leaves took place. At the lower part of the bromeliads, where the leaves are already growing densely clustered, rigidity was maintained, and no further bending was possible [**S4 Table in** S1 File]. Thus, the limited flexibility of these epiphytes makes them more comparable to trees and produced similar *C*_*d*_ values: trees at equivalent wind speeds show *C*_*d*_ values of 0.6–0.7 [64].

Our results are also interesting in regard to the Vogel exponent, given that currently values are mainly available for aquatic plants [see Table 1 in 51]. Theoretically, the Vogel exponent of most plants should fall close to -2/3 because the reconfiguration of most plants is similar to that of the bending of a single beam or a rectangular plate, where the simple bending results in the loss of just one characteristic length in the system during reconfiguration [48]. However, this was not observed in bromeliads. The shape-change of these bromeliads with increasing wind velocity was more complex than a single bending beam as observed in grasses or in individual shoots. Bromeliads reconfigured their bodies by aligning the leaves in the flow direction, and as wind velocities increased, leaves clustered and effectively reduced the overall plant frontal area. Consequently, the Vogel exponent was clearly not constant over the tested wind velocities but changed in a stepwise fashion (Fig 6). For example, in *G*. *monostachia*, at wind speeds of c. 9 m s^-1^, the drag-velocity relationship changed from a quadratic relationship (*B* = -0.53; *F*_*d*_ ~ *v*^1.47^) to an almost linear one (*B* = -0.98; *F*_*d*_ ~ *v*^1.02^) (Fig 6).

Flexible, sessile organisms exposed to flows of water or air are able to undergo some form of reconfiguration due to their structural flexibility [37]. Flexibility of the drag body does play a role in the adaptive reconfiguration to minimise drag in flows [65–67]. Although no mechanical tests were carried out in this study to investigate the flexibility of the plants, it can be indirectly inferred from the Vogel exponent. When a plant has more flexibility to twist and reconfigure in the wind flow, it achieves greater relative reduction of drag as speed increases, and this is reflected by a lower (more negative) Vogel exponent [see 65, Fig 4, the more flexible plate had the greatest relative reduction of drag]. In this study, *Guzmania monostachia* was able to achieve greater reconfiguration than the other tested species, as reflected by the Vogel exponent. Even at the lower wind speeds, before the sudden change in the slope of the regression line, the mean Vogel exponent for *G*. *monostachia* was lower than the other species (Table 2, *B* = -0.45). In contrast, the mean Vogel exponents for *T*. *fasciculata* and *T*. *flexuosa* were the highest (less negative) (Table 2, slope 2), suggesting that they were the least flexible and less effective in achieving drag reduction by reconfiguration. Moreover, these two species have stiffer leaves than the other species [pers. obs., **S4 Table in** S1 File]. Therefore, the higher Vogel exponents from these two species were not surprising. Since more negative Vogel exponents indicate greater ability to streamline, our results highlight the limited reconfiguration capacity in certain species of bromeliads that prevents the plant from achieving a more streamlined shape at higher wind velocities without sustaining morphological damage. There was no clear pattern regarding the threshold wind velocity at which the plants reconfigured into a more streamlined shape. The way the plants behaved in wind flow depended on the nature and mechanical properties of each individual plant.

### 4.1 Mechanical loading in the ecological context

**Fig 7:**
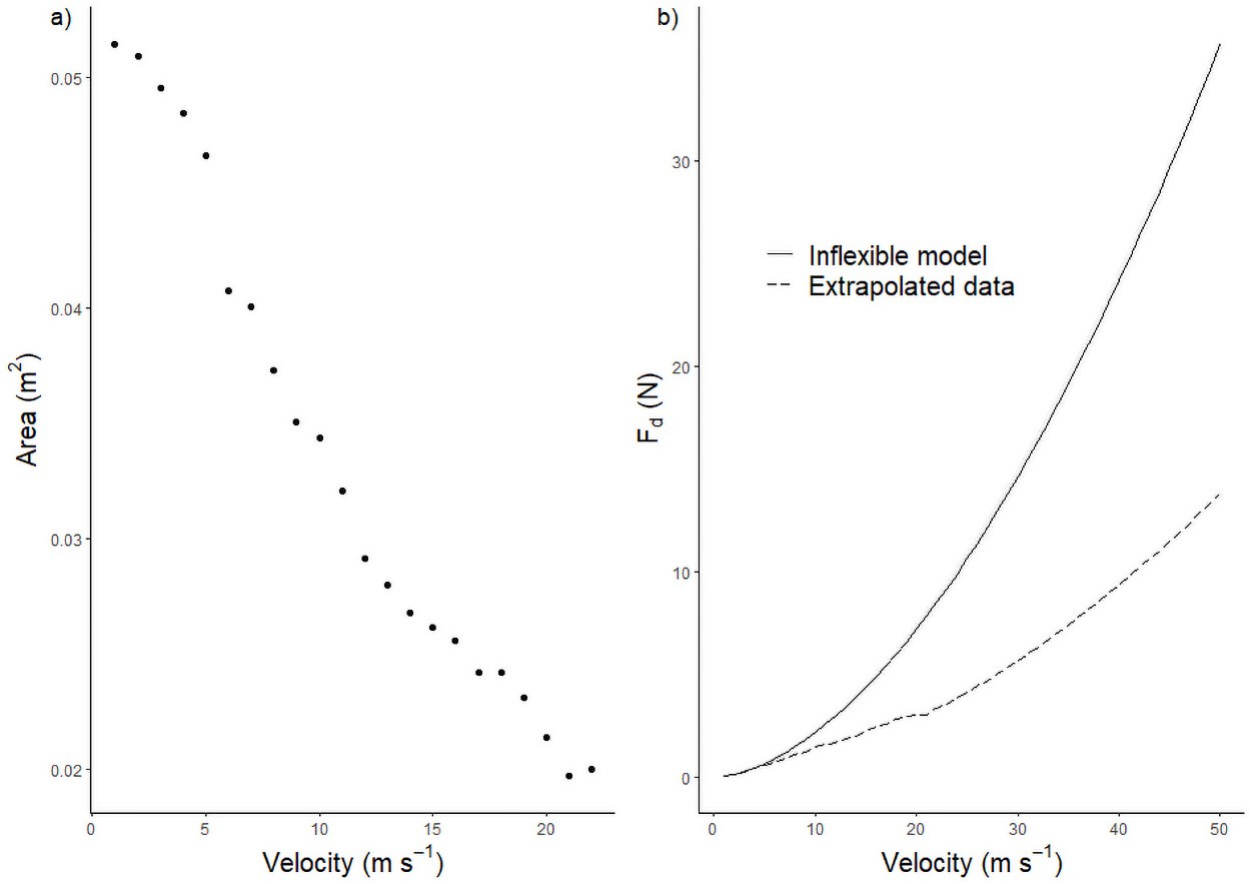
Frontal area reduction of one individual and the associated extrapolated reduction in drag forces. a) Decrease in the frontal area of an individual of G. monostachia (size: 35 cm) over increasing wind velocity. The rate of area reduction was decreasing and almost reaching a constant which was later used for extrapolation. b) Theoretical model showing the extent of drag force reduction on the individual of G. monostachia, assuming the frontal area of the plant stopped decreasing as plant reached maximum moment of bending as seen in a).

The majority of the bromeliads used in this study were collected from *Annona glabra* trees growing on the lake in the areas around Barro Colorado Island. These trees are small statured and have a relatively open crown structure that hosts a thriving epiphyte community [68]. During the dry months of January to April, the northeast trade wind brings the highest wind speeds to the area [69]. The highest long-term average was recorded to be c. 2.7 m s^-1^ in February and the long-term average maximum for that month is c. 8.6 m s^-1^ [70]. These data were collected on a meteorological tower at 48 meters, which is above the forest canopy. Therefore, within the forest, lower wind speeds are expected. Epiphytes growing on the *Annona glabra* trees that are located on the fringe of the island may experience more wind than those growing on trees within the forest. Although we do not have wind speed data from the *Annona glabra* trees, the measurements from the meteorological tower may be a good approximation. As shown in this study, none of the individuals exposed to wind speeds of up to 22 m s^-1^ showed any physical damage and recovered their original form immediately. From our results, especially so for larger individuals, drag reduction by reconfiguration against the wind speeds tested in this experiment proved to be an effective mechanism. While the wind velocities tested in this study are below “Typhoon” or “Hurricane” grade, i.e., > 33 m s^-1^, a theoretical reduction in drag forces at those wind speeds can be extrapolated from our results (Fig 7). For example, the drag reduction as compared to an inflexible model for a *G*. *monostachia* individual, at velocity 10, 20 and 50m s^-1^, would be 33, 58 and 61%, respectively (Fig 7b). Although this illustrates that reconfiguration is a robust and effective passive process for load reduction, it is naïve to assume that the same would be observed in nature, because a plant would hardly remain undamaged at very high wind velocities. There is direct evidence of physical damage as leaves were torn when large, broad-leaved bromeliads, like *V*. *sanguinolenta*, were exposed to wind speeds of 30–50 m s^-1^ (HJR Einzmann, unpubl. res.). Moreover, the difference in the drag reduction between flexible plant and inflexible model for the wind velocities of 20 and 50 m s^-1^, was only 3%. Thus, at very high flow velocities, drag reduction by reconfiguration is likely to lose its effectiveness and the plant would probably be damaged.

Wind tunnel experiments allow an in-depth understanding of the reconfiguration mechanism to reduce drag loading in bromeliads in wind flow. However, the laminar flow used in this study is not a realistic representation of the natural dynamics of wind flow in forests, since the drag coefficient is also influenced by turbulence [71, 72]. Wind flow in a natural forest is highly turbulent, which exerts irregular loads on branches and can result in tree damage [73]. On the other hand, epiphyte-laden branches are often swaying with the wind (HJR Einzmann, ‘pers. obs.’). Unpublished video recordings reveal hardly any movements of leaves of the bromeliads. Thus, branch movements may further offset the overall wind loading acting on the bromeliad, as compared to the fixed position of a plant in the wind tunnel. In the natural environment, the streamlining process of bromeliads is probably more irregular due to the turbulent wind flow in contrast to laminar flow in the wind tunnel.

In this study, individual bromeliads were subjected to wind without their host plant, a scenario that is never occurring in nature. On the tree, an epiphyte might grow in a cluster together with other plants experiencing interactions such as additional frictional contact with neighbouring plants. Drag forces experienced under this scenario will be very different from our results. We did not consider those interactions—our approach resembles potential drag force reduction by reconfiguration of epiphytes on trees with exposed, sparsely vegetated branches. For epiphytes located inside the crown, they are mostly sheltered from the wind by the foliage of the tree. The main drag force from wind acts probably on the whole epiphyte-host system. In nature, bromeliads may also have a functional tank that is filled with organic matter and water, adding to the overall weight of the plant. Although drag force is not a function of mass, this additional weight would ultimately increase the total mechanical wind loading on the branch where the plant is attached to. Acknowledging all these caveats, this study is still an important first step towards understanding the mechanisms of reconfiguration by epiphytes in wind by quantifying the forces bromeliads face in a wind stream. As such, future drag force testing should include measurement of drag on the whole epiphyte-branch system, to investigate how total drag force on the host can be affected by the presence of epiphytes on its branches and/or trunk. Tests to determine flexural rigidity, modulus of elasticity and Cauchy number could be included in future experiments for more realistic understanding of the complex drag force of flexible vegetation [47]. Our study quantifies the drag force from wind loading on bromeliads, but there is very limited knowledge on the actual forces needed to dislodge bromeliads from their substrate. Such information would be essential to understand under which conditions epiphytes face the risk of dislodgement in storm and windy events.

## Supporting information

S1 File (DOCX)
