# Supplementary material for: Go with the flow: The extent of drag reduction as epiphytic bromeliads reorient in wind
Source: PLoS One. 2021 Jun 24;16(6):e0252790. doi: 10.1371/journal.pone.0252790 (PMC8224879; doi:10.1371/journal.pone.0252790)
Supplement: S1 File — (DOCX) [file pone.0252790.s001.docx]

S1 File

Title: Go with the flow: the extent of drag reduction as epiphytic bromeliads reorient in wind

Authors: Jessica Y. L. Tay^1, *^, Gerhard Zotz^1,3^, Jaroslaw Puczylowski^2^, Helena J. R. Einzmann^1^

^1^ Functional Ecology of Plants, Institute of Biology and Environmental Sciences, University of Oldenburg, Oldenburg, Germany

^2^ ForWind – Center for Wind Energy Research, University of Oldenburg, Oldenburg, Germany

^3^ Smithsonian Tropical Research Institute, Panama, Republic of Panama

*Corresponding author

Email: [jessica.tay.ying.ling@uni-oldenburg.de](mailto:jessica.tay.ying.ling@uni-oldenburg.de)

Table S1: Selected studies on the forest damage caused by tropical storms in the last 40 years, i.e., 1969 – 2019.

| Storm | Study location | Date | Max. winds (km/h) | Damages | Reference |
| --- | --- | --- | --- | --- | --- |
| Hurricane David | Forests (including rainforest, lower montane forest, dry scrub woodlands, urban and secondary forest) of Dominica, eastern Caribbean | August 29, 1979 | 241 | 3% and 6% loss in basal area in rainforest and lower montane forest respectively; 42% damage in commercial timber | Lugo, Applefield [1] |
| Hurricane Gilbert | Lower montane wet forests, Blue mountains, Jamaica | September 12, 1988 | 255 | 8.1 ± 1.4% Stem mortality; Low overall damage in the study area of 2.5km^2^ | Bellingham [2] |
| Hurricane Joan | Pine forest and broad-leaved rainforest, Southeastern coast, Nicaragua | October 22, 1988 | 250 | 58% and 12.8% tree mortality in a pine forest and rainforest, respectively | Boucher, Vandermeer [3] |
| Hurricane Hugo | Lower montane rainforest, semi-evergreen forest and mangrove forest, Guadeloupe | September 16, 1989 | 230; wind gusts, 296 | 13%, 10% and 41% decrease in stem density in lower montane rainforest, semi-evergreen and mangrove forest, respectively  10%, 12% and 37% decrease in basal area in lower montane rainforest, semi-evergreen and mangrove forest, respectively | Imbert, Labbe [4] |
| Hurricane Andrew | Mangrove forest, south Florida | August 24, 1992 | 232;  wind gust, 280 | 85.1% mortality of three mangrove species | Baldwin, Egnotovich [5] |
| Hurricane | Mixed hardwood forests in Mingo national Wildlife Refuge, Missouri | June 4, 1993 | No info | 30% individual trees, 64% basal area blown down; 20% trees died | Peterson and Rebertus [6] |
| Typhoon Paka | *Cycas micronesica* population, Guam | December 16, 1997 | 241; wind gusts, 297 | 65% trees with partial canopy damage; 10% trees with complete defoliation, 10% trees with decapitated canopy, <2% fallen trees | Hirsh and Marier [7] |
| Typhoon Haiyan | Mangrove forest, Eastern Samar, Philippines | November 8, 2013 | 251;  wind gusts,  > 300 | 20.4% mangrove area impacted; 870 hectares severely damaged. | Long, Giri [8] |
| Tropical Cyclone Lionrock | Korean Pine-broadleaved forest, Russian Far East | August 31, 2016 | 108 | More than 400km^2^ of windthrow patches; in one contiguous patch, tree mortality of 54%. | Vozmishcheva, Bondarchuk [9] |

Table S2: List of publications in the primary literature with observations of effects of storms on epiphytes and host. Superscripts with storm names indicate the hurricane category (5: peak sustained wind speeds > 252 km h^-1^, 4: peak sustained wind speeds > 209 km h^-1^).

| Event | Country | Aspect | Method | Taxa | Main result | Publication |
| --- | --- | --- | --- | --- | --- | --- |
| Hurricane Hugo^4^ | Puerto Rico | Plant-pollinator interaction | Field observation | 1 orchid and 1 hawk moth | Severe habitat alteration but minimal short-term consequences from hurricane | Ackerman and Moya [10] |
| Hurricane Mitch^5^ | Honduras | Community effect | Census | Many species | Effects still detectable 14 years after the last hurricane | Batke and Kelly [11] |
| Hurricane Andrew^5^ | Florida | Immediate damage | Field observation | Several species | Survivors with fruit “probably rebuild populations” | Benzing [12] |
| Hurricane Donna^5^ | Florida | Immediate damage | Quantification of damage | Many species | 50-90% of all epiphytes destroyed | Craighead and Gilbert [13] |
| Hurricane Hugo^5^ | Puerto Rico | Impact of epiphytes on host tree | Field observation | n/a | resistance to wind in older individuals with extensive epiphyte growth causes tree falls | Frangi and Lugo [14] |
| Hurricane Wilma^5^ | Mexico | Immediate damage | Field observation | Many species | > 50 % of all epiphytes destroyed | Goode and Allen [15] |
| Hurricane George^4^ | Puerto Rico | Demography after transplantation | Matrix analysis post hurricane | 1 orchid species | Transplantation as potential conservation tool | Joubert and Tremblay [16] |
| Hurricane Andrew^5^ | Florida | Immediate damage | Field observation | Many species | 90% of all canopy epiphytes destroyed | Loope, Duever [17] |
| "no-name storm" | Florida | Mortality after storm | Field observation | 4 species | Mortality c. 40% | Lowman and Linnerooth [18] |
| typhoon Paka^5^ | Guam | Impact of epiphytes on host tree | Field observation | Epiphytic ferns | Increased resistance to wind promotes snapping of trees | Marler and Lawrence [19] |
| unspecified | Japan | Demography after disturbance | Matrix analysis post hurricane | 1 orchid species | Successful recovery | Matsumura and Sawada [20] |
| Hurricane Hugo^5^ | Puerto Rico | Immediate damage | Field observation | 11 orchid species | Complete eradication during the storm | Migenis and Ackerman [21] |
| Hurricane Ivan^5^ | Cuba | Demography after disturbance | Matrix analysis post hurricane | 2 orchid species | Partial recovery in subsequent years | Mújica, Raventós [22] |
| Hurricane Andrew^5^ | Florida | Damage and recovery | Inventories pre and post hurricane | 5 bromeliad species | Post-storm recruitment compensates for immediate damage | Oberbauer, Whelan [23] |
| Hurricane Ivan^5^ | Cuba | Population dynamics | Pre- and post- hurricane dataset | 1 orchid species | Severe impact; annual population decline of 14% | Ortiz-Rodríguez, Raventós [24] |
| unspecified | Puerto Rico | Survival after dislodgement | Experimental transplantation | 1 bromeliad species | Relatively high survival on the ground | Pett‐Ridge and Silver [25] |
| Hurricane Ivan^5^ | Cuba | Viability analysis | Field observation | 2 orchid species | Possible necessity of management to counter negative population trends | Raventós, González [26] |
| Hurricane Ivan^5^ | Cuba | Demography after disturbance | Field observation | 1 orchid species | High frequency of hurricanes leads to extinction | Raventós, González [27] |
| Hurricane Andrew^5^ | Florida | Recovery | Field observation | 4 bromeliad species | Good recovery in the five years after the hurricane | Robertson and Platt [28] |
| Hurricane Hugo^5^ | Puerto Rico | Immediate damage | Field observation | 1 orchid species | 30% of the population either destroyed or damaged | Rodríguez-Robles, Ackerman [29] |
| Hurricane Dean^5^ | Martinique | Demography after disturbance | Census | Many climbers and hemiepiphytes | Number of climbers and hemiepiphytes declined by 9% | Schnitzler, Arnold [30] |
| Hurricane George^4^ | Puerto Rico | Survival after relocation | Field observation | 1 orchid species | Transplantation as potential conservation tool | Tremblay [31] |
| several hurricanes^5^ | Cuba | Demography | Field observation | 1 orchid species | Reductions in population size due to several factors, one of which is hurricane damage | Vale, Rojas [32] |
| Hurricane Hugo^5^ | Puerto Rico | Damage and recovery | Field observation | Many species | Uneven recovery after the storm | Weaver [33] |
| Hurricane Ivan^5^ | Cuba | Recovery | Field observation | 2 orchid species | Partial recovery in subsequent years | Wiegand, Raventós [34] |


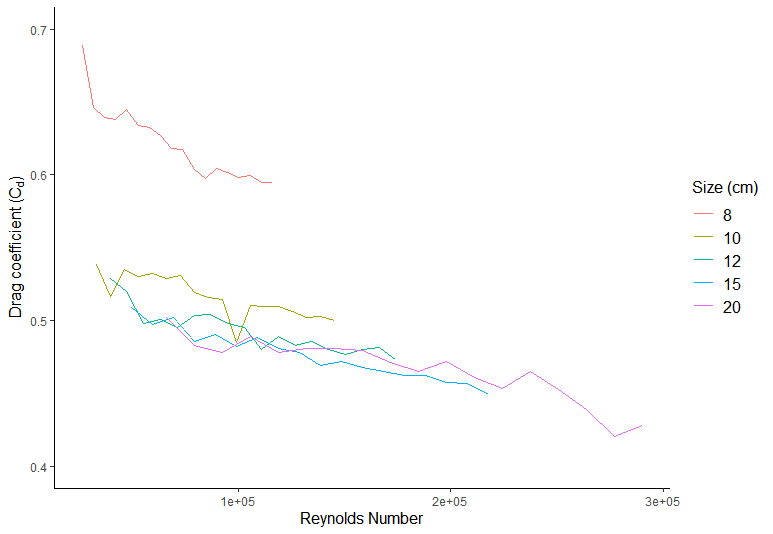


**Fig S1. Drag coefficients of spheres varying in size as a function of Reynolds number**

The C_d_ values of the spheres were around 0.43 – 0.54, with the exception of the 8 cm sphere. Once the diameter of the sphere gets too small relative to the size of the support pole behind it, the dominant shape is no longer spherical, resulting in a different overall drag body shape. None of the tested plants used in the experiment had a length ≤ 8 cm. Acknowledging this caveat, the wind tunnel setup generated reliable experimental data and was suitable for further investigation with the collected epiphytes.


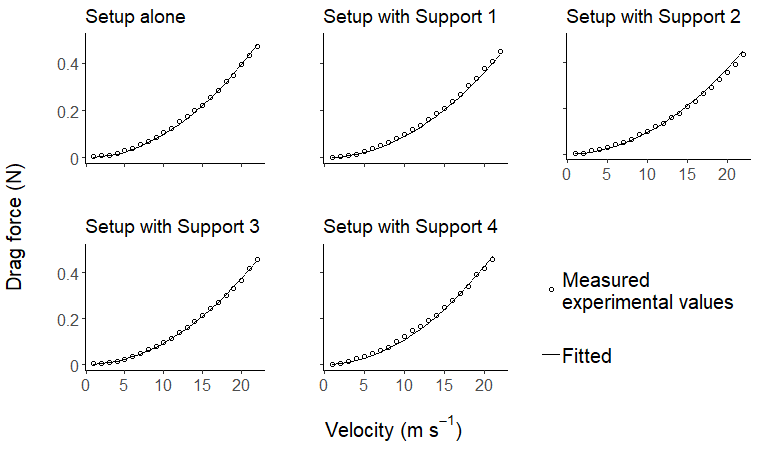


**Fig S2. Drag force exerted on the wind tunnel setup**

Various 3D-printed supporting cylinders were used to hold each bromeliad in place, whenever required. The diameter (cm) and height (cm) of each supporting cylinder are as follow: Support_1 = 1 cm x 2 cm; Support_2 = 1.5 cm x 2 cm; Support_3 = 2 cm x 2 cm; Support_4 = 2.5 cm x 3 cm. Larger supports were used for larger plants. Fitted values were modelled based on the data from 8 – 22 m s^-1^, because measurements below 8 m s^-1^ were < 0.05 N due to the small size of the setup. These offset values were subsequently subtracted from the total drag force of each measurement for each sample to ensure that recorded drag force was on the sample alone. Nonetheless, the measured experimental values followed the fitted model well.

Text 1: Detailed procedure to create stiffened plants

First, the plants were coated with a layer of watered-down white PVA glue (Holzleim Classic, Ponal, Germany) and let to dry. The coating with white glue was necessary because it created a temporary protective layer against the slightly exothermic reaction of the epoxy resin. Next, plants were coated with one layer of epoxy resin which was mixed as instructed, 10 ml resin to 6 ml hardener (Yachtcare Epoxy laminate with hardener, Vosschemie, Uetersen, Germany). Once dry, another layer of epoxy resin mixed with fibre glass (16 ml epoxy resin mixture to 1 g fibre glass; Screed glass fibre, 12 mm fibre length) was coated on the plants for extra stiffness. Longer leaves that were still drooping after the fibre glass coating were additionally supported with wires.

Table S3. Schematic reduction in frontal areas for varying sizes of G. monostachia. Plant sizes was based on the longest leaf length of the individual plant. Numbers in white boxes are the frontal area of the individual plant at the given wind speed. Area reduction was based on the overall decrease in frontal area of the plant between the lowest and highest wind speeds. Drag force reduction was based on the decreased in drag on the plant at the highest wind speed (i.e., 22 m s^-1^), compared to the stiff model. Frontal area reduction is greater in large plants compared to that of smaller plants. Given that the same fitted function of C_d_(v) was used for these individuals (Fig 3), the decrease in frontal area was clearly the main factor resulting in drag force reduction.

| Plant size (cm)  Wind  Speed (m s^-1^) | 35.3 | 23.4 | 15.2 |
| --- | --- | --- | --- |
| 5 | 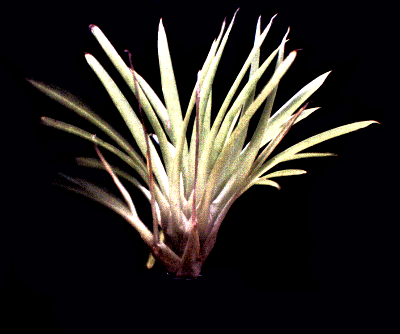  0.047 m^2^ | 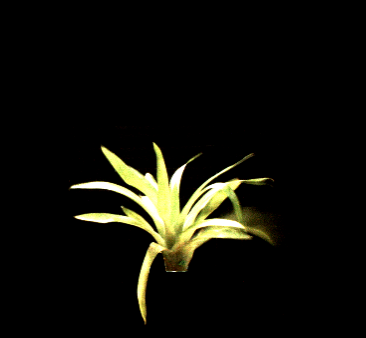  0.014 m^2^ | 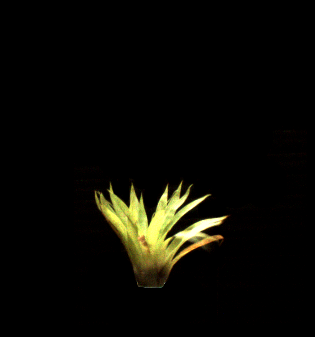  0.007 m^2^ |
| 10 | 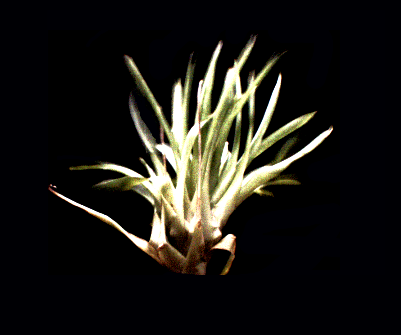  0.034 m^2^ | 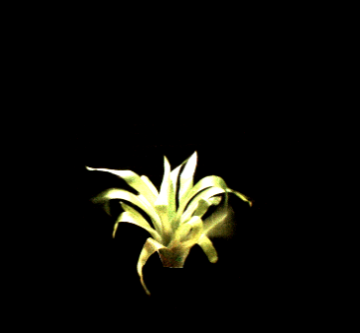  0.013 m^2^ | 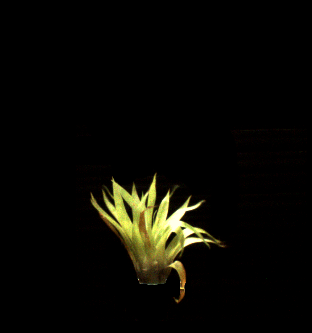  0.007 m^2^ |
| 15 | 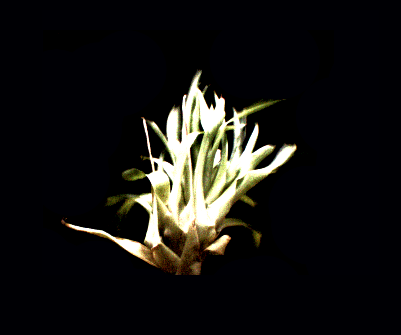  0.026 m^2^ | 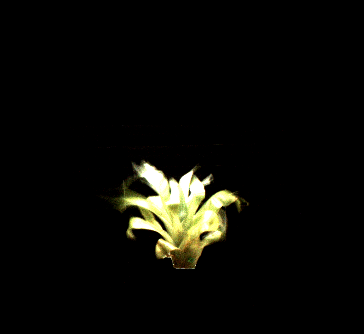  0.010 m^2^ | 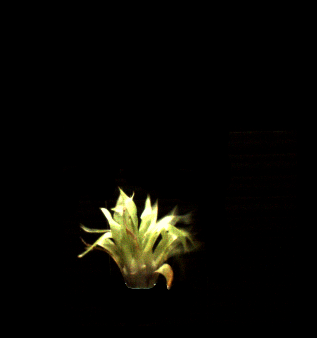  0.006 m^2^ |
| 22 | 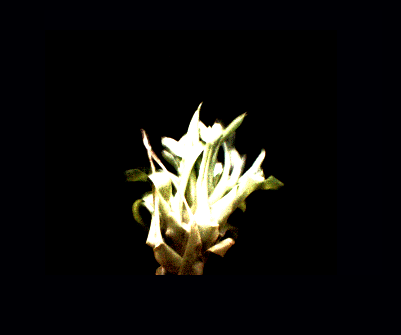  0.020 m^2^ | 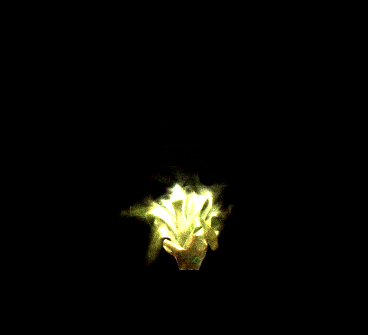  0.008 m^2^ | 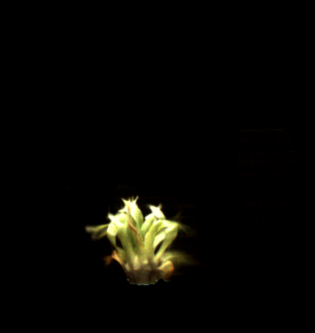  0.005 m^2^ |
| Area reduction (%) | 57 | 43 | 29 |
| Drag force reduction (%) | 62 | 35 | 12 |

Table S4: Photographic representation of foliage reconfiguration of all five species. These photographs were taken by the camera installed at the side of the setup. Each species is represented by the smallest and the largest collected individuals, based on the longest leaf. As wind velocity increased, leaves started to bend and cluster in the direction of flow. At the highest wind velocity, the rigidity of the lower part of the bromeliads was maintained and no further bending was observed. Smaller plants, with their shorter leaves had little leeway for leaf reconfiguration, hence they did not reduce drag as effectively as larger plants.

| Wind speed (m s^-1^) | 5 | 10 | 15 | 22 |
| --- | --- | --- | --- | --- |
| *G. monostachia*  Size: 35 cm | 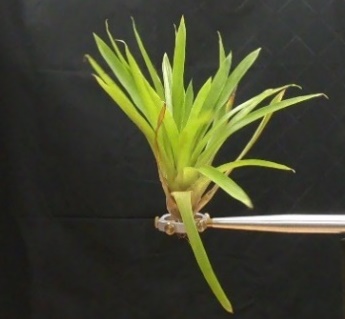 | 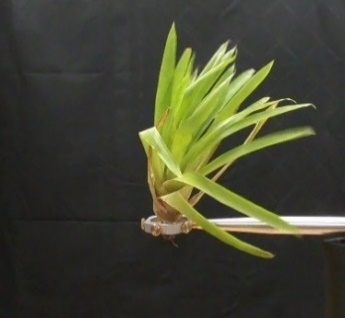 | 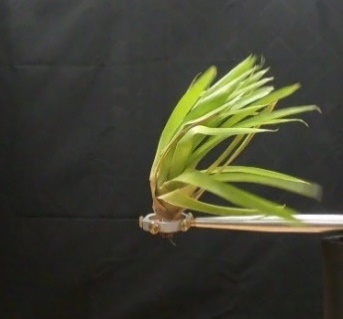 | 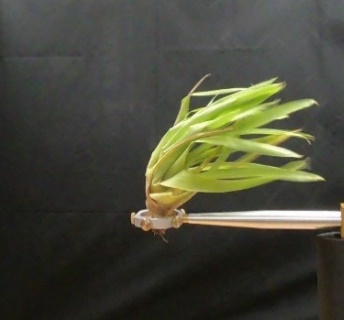 |
| *G. monostachia*  Size: 15 cm | 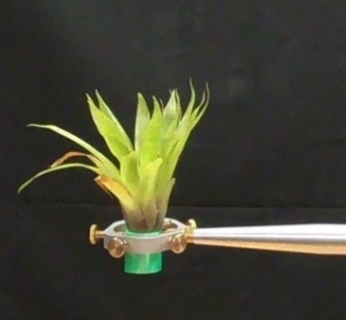 | 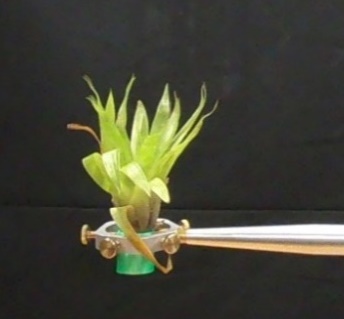 | 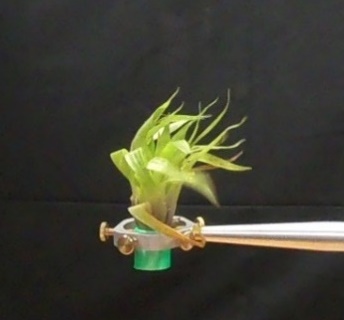 | 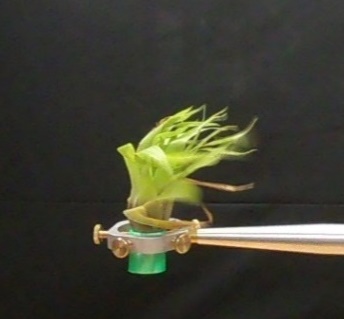 |
| *T. elongata*  Size: 41 cm | 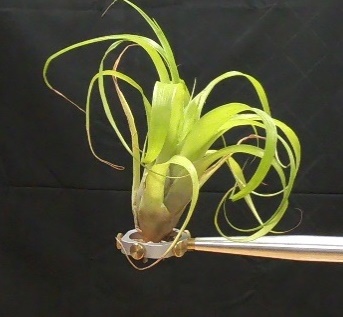 | 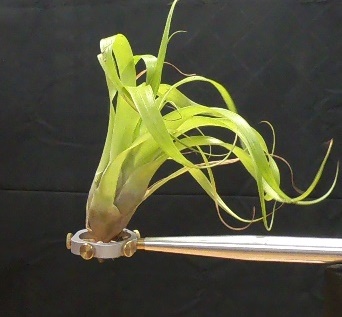 | 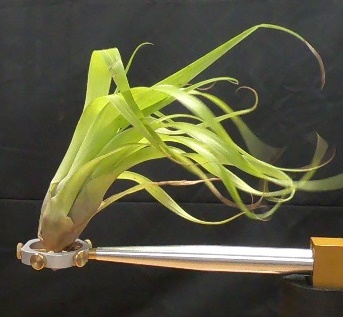 | 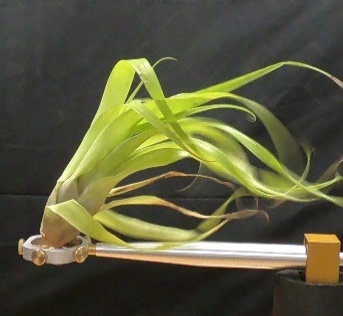 |
| *T. elongata*  Size: 17 cm | 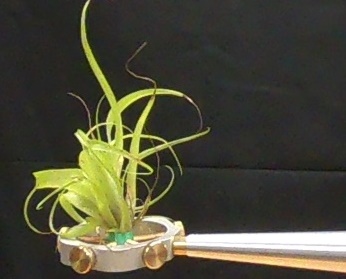 | 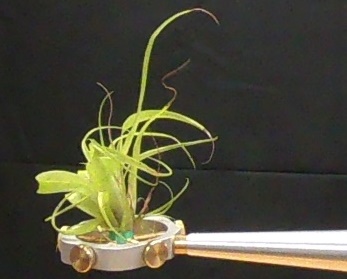 | 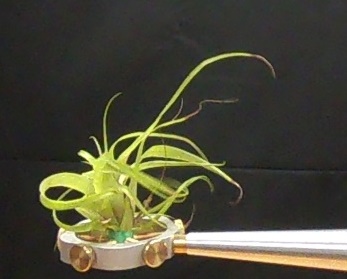 | 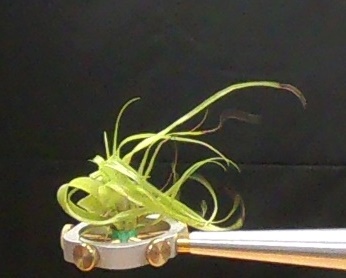 |
| *T. fasciculata*  Size: 49 cm | 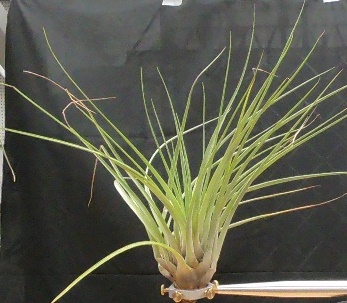 | 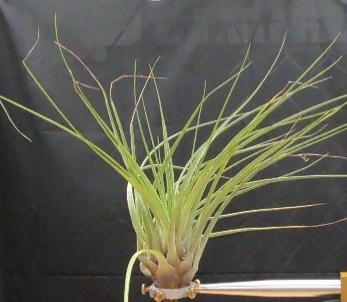 | 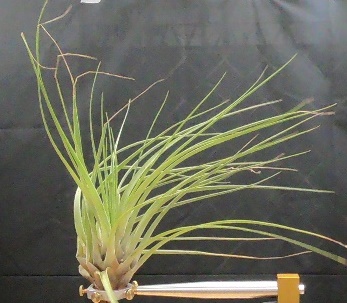 | 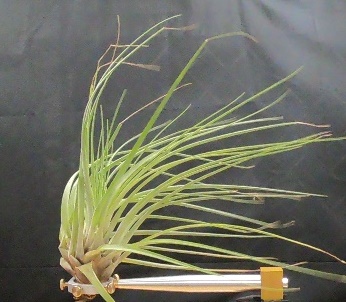 |
| *T. fasciculata*  Size: 13 cm | 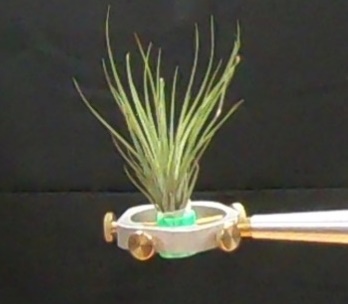 | 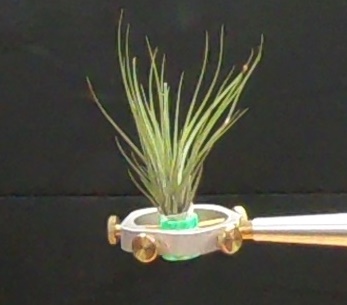 | 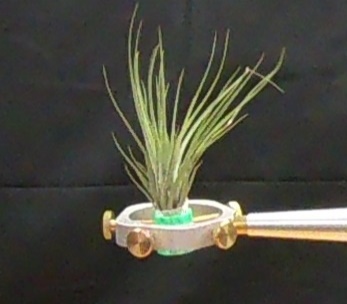 | 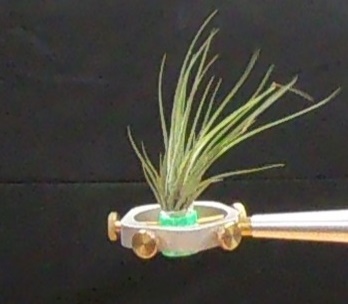 |
| *V. sanguinolenta*  Size: 47 cm | 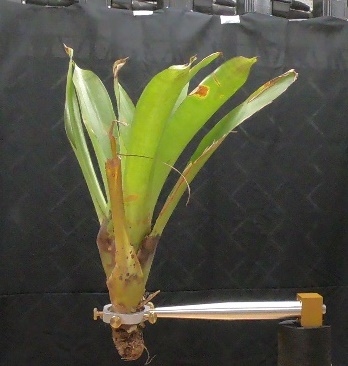 | 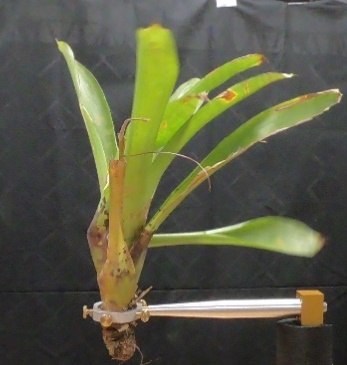 | 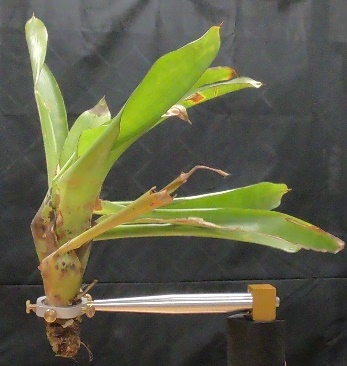 | 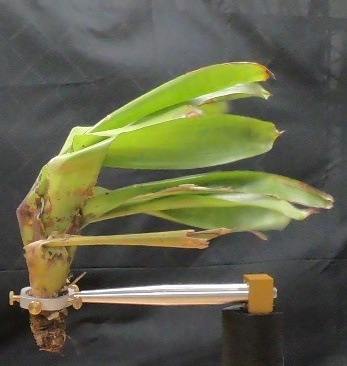 |
| *V. sanguinolenta*  Size: 16 cm | 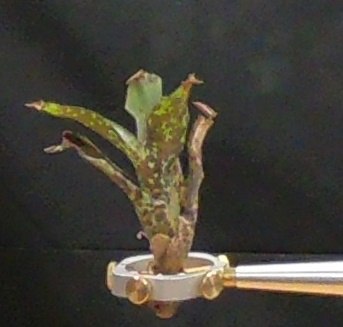 | 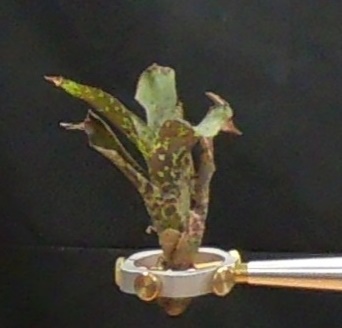 | 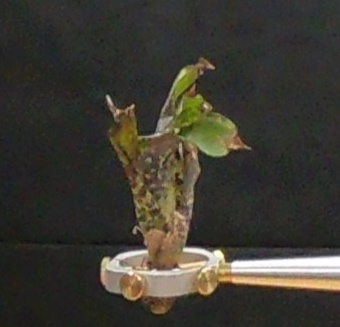 | 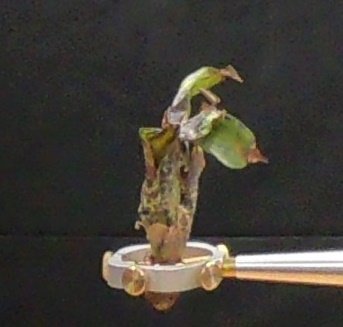 |
| *T. flexuosa* Size: 49 cm | 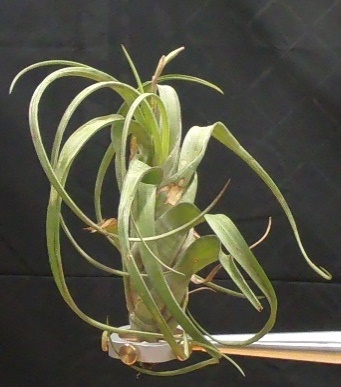 | 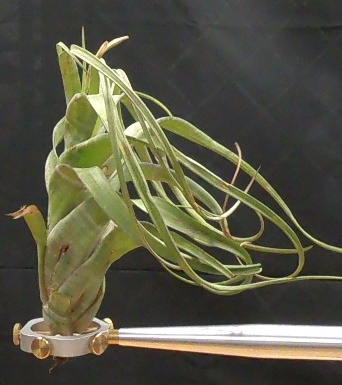 | 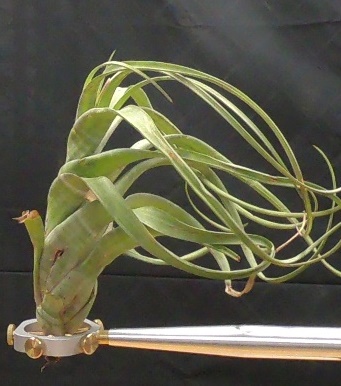 | 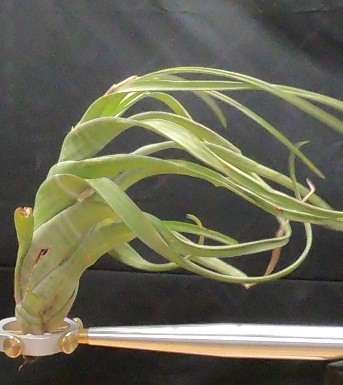 |
| *T. flexuosa* Size: 13 cm | 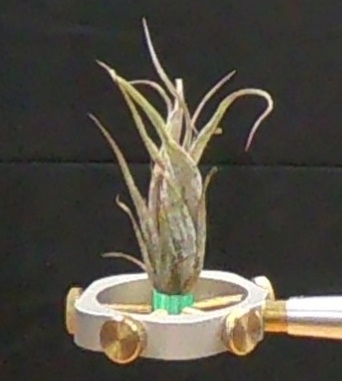 | 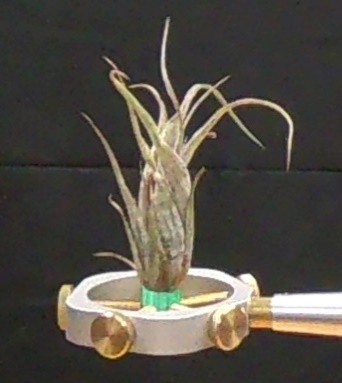 | 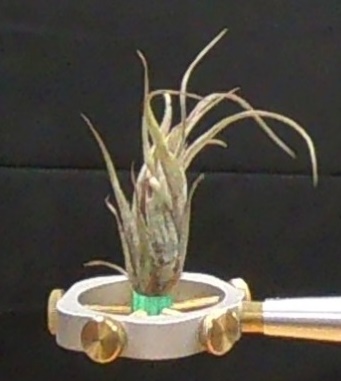 | 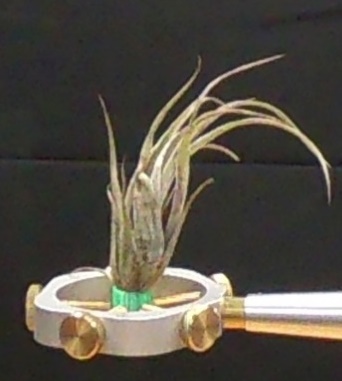 |


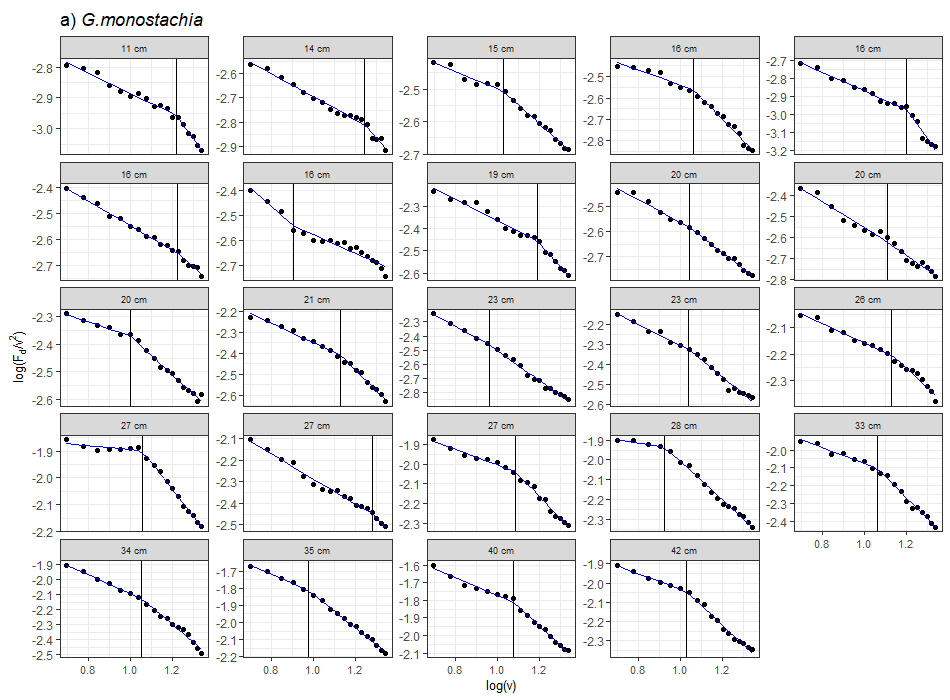

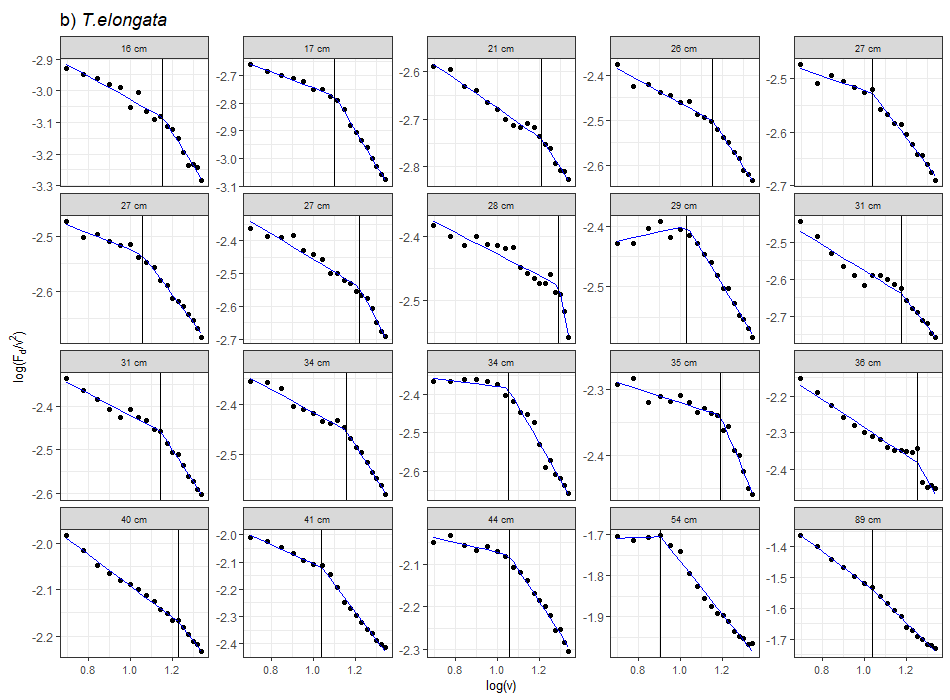

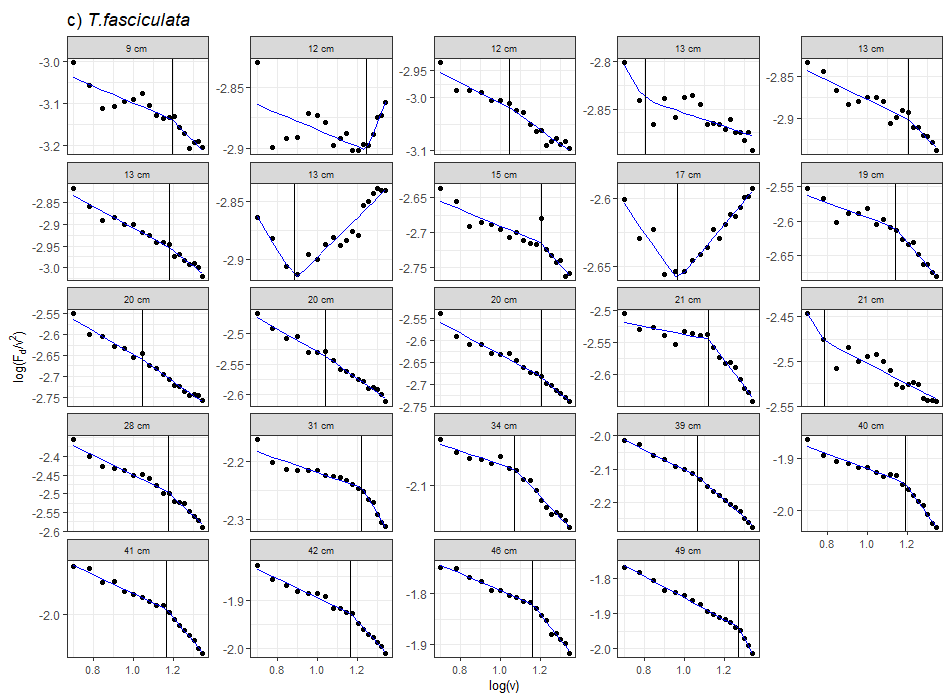

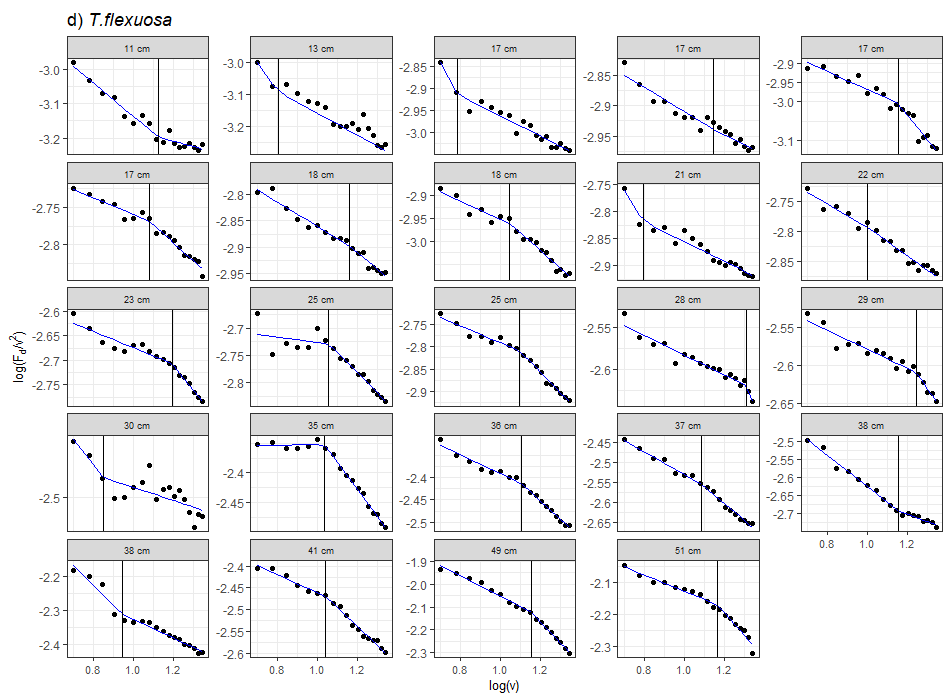

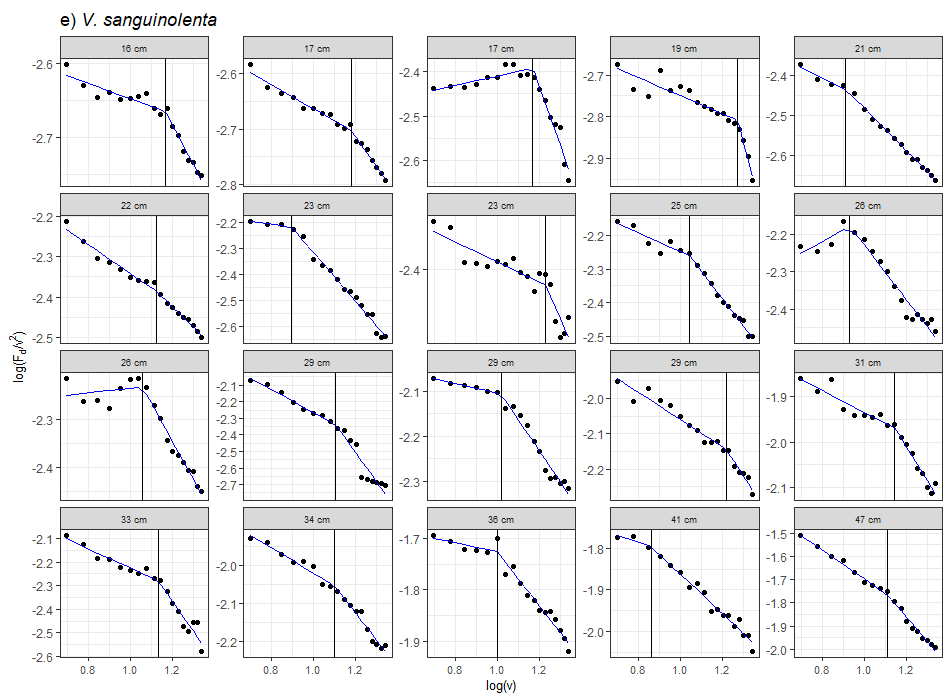


Figure S3: Vogel exponents (slope of the regression line) of all individuals of all species. Plots are arranged in ascending order in terms of plant size for each species. a) G. monostachia, b) T. elongata. c) T. fasciculata, d) T. flexuosa, e) V. sanguinolenta. Labels in the grey boxes refer to the LL of an individual. The vertical line indicates the breakpoint where the slope of the regression line changed. Generally, the slope increased after the breakpoint, especially for larger individuals. This means that at higher wind velocity, drag force increased more linearly with wind speed, deviating from the quadratic relationship.

Literature cited in the supplement

1. Lugo AE, Applefield M, Pool DJ, McDonald RB. The impact of Hurricane David on the forests of Dominica. Can. J. For. Res. 1983;13(2):201-11. doi: 10.1139/x83-029.

2. Bellingham P. Landforms influence patterns of hurricane damage: evidence from Jamaican montane forests. Biotropica. 1991;23(4a):427-33. doi: 10.2307/2388262.

3. Boucher DH, Vandermeer JH, Yih K, Zamora N. Contrasting hurricane damage in tropical rain forest and pine forest. Ecology. 1990;71(5):2022-4. doi: 10.2307/1937611.

4. Imbert D, Labbe P, Rousteau A. Hurricane damage and forest structure in Guadeloupe, French West Indies. J. Trop. Ecol. 1996;12(5):663-80. doi: 10.1017/S026646740000986X.

5. Baldwin A, Egnotovich M, Ford M, Platt W. Regeneration in fringe mangrove forests damaged by Hurricane Andrew. Plant Ecol. 2001;157(2):151-64. doi: 10.1023/A:1013941304875.

6. Peterson CJ, Rebertus AJ. Tornado damage and initial recovery in three adjacent, lowland temperate forests in Missouri. J. Veg. Sci. 1997;8(4):559-64. doi: 10.2307/3237207.

7. Hirsh H, Marier T. Damage and recovery of *Cycas micronesica* after Typhoon Paka. Biotropica. 2002;34(4):598-602. doi: 10.1111/j.1744-7429.2002.tb00579.x.

8. Long J, Giri C, Primavera J, Trivedi M. Damage and recovery assessment of the Philippines' mangroves following Super Typhoon Haiyan. Mar. Pollut. Bull. 2016;109(2):734-43. doi: 10.1016/j.marpolbul.2016.06.080.

9. Vozmishcheva AS, Bondarchuk SN, Gromyko MN, Kislov DE, Pimenova EA, Salo MA, et al. Strong Disturbance Impact of Tropical Cyclone Lionrock (2016) on Korean Pine-Broadleaved Forest in the Middle Sikhote-Alin Mountain Range, Russian Far East. Forests. 2019;10(11):1017. doi: 10.3390/f10111017.

10. Ackerman JD, Moya S. Hurricane aftermath: resiliency of an orchid-pollinator interaction in Puerto Rico. Caribb. J. Sci. 1996;32:369-74.

11. Batke SP, Kelly DL. Changes in the distribution of mechanically dependent plants along a gradient of past hurricane impact. AoB Plants. 2015;7:plv096. doi: 10.1093/aobpla/plv096.

12. Benzing DH. Bromeliaceae: profile of an adaptive radiation: Cambridge University Press; 2000.

13. Craighead FC, Gilbert VC. The effects of Hurricane Donna on the vegetation of southern Florida. Q. J. Fla. Acad. Sci. 1962;25(1):1-28.

14. Frangi JL, Lugo AE. Hurricane damage to a flood plain forest in the Luquillo Mountains of Puerto Rico. Biotropica. 1991;23(4a):324-35. doi: 10.2307/2388248

15. Goode LK, Allen MF. The impacts of Hurricane Wilma on the epiphytes of El Edén Ecological Reserve, Quintana Roo, Mexico. J. Torrey Bot. Soc. 2008;135(3):377-88. doi: 10.3159/07-RA-052.1.

16. Joubert RJB, Tremblay RL. Efecto de remoción y relocalización de *Lepanthes eltoroensis* Stimson, despues de un Huracán. Lankesteriana. 2003;7:67-70. doi: 10.15517/lank.v3i2.23019.

17. Loope L, Duever M, Herndon A, Snyder J, Jansen D. Hurricane impact on uplands and freshwater swamp forest. Bioscience. 1994;44(4):238. doi: 10.2307/1312228

18. Lowman M, Linnerooth W. Population dynamics of some native Florida epiphytes. II. Mortality after a storm. Journal of the Bromeliad Society. 1995;45(1):15-7.

19. Marler TE, Lawrence JH. Phytophagous insects reduce cycad resistance to tropical cyclone winds and impair storm recovery. HortScience. 2013;48(10):1224-6. doi: 10.21273/HORTSCI.48.10.1224.

20. Matsumura T, Sawada Y. Demography of the epiphytic orchid *Sarcochilus japonicus*, as assessed on the basis of the fallen trees disturbed by a windstorm. Veg. Sci. 2009;26(2):103-10. doi: 10.15031/vegsci.26.103.

21. Migenis LE, Ackerman JD. Orchid-phorophyte relationships in a forest watershed in Puerto Rico. J. Trop. Ecol. 1993;9(2):231-40. doi: 10.1017/S0266467400007227.

22. Mújica E, Raventós J, González E, Bonet A. Long-term hurricane effects on populations of two epiphytic orchid species from Guanahacabibes Peninsula, Cuba. Lankesteriana. 2013;13(1-2):47-55. doi: 10.15517/lank.v0i0.11534.

23. Oberbauer SF, Whelan KR, Koptur S. Effects of Hurricane Andrew on epiphyte communities within cypress domes of Everglades National Park. Ecology. 1996;77(3):964-7. doi: 10.2307/2265516

24. Ortiz-Rodríguez IA, Raventós J, Mújica E, González-Hernández E, Vega-Peña E, Ortega-Larrocea P, et al. Spatiotemporal effects of Hurricane Ivan on an endemic epiphytic orchid: 10 years of follow-up. Plant Ecol. Divers. 2019;12:1-17. doi: 0.1080/17550874.2019.1673495.

25. Pett‐Ridge J, Silver WL. Survival, growth, and ecosystem dynamics of displaced bromeliads in a montane tropical forest. Biotropica. 2002;34(2):211-24. doi: 10.1111/j.1744-7429.2002.tb00532.x.

26. Raventós J, González E, Mújica E, Bonet A. Transient population dynamics of two epiphytic orchid species after Hurricane Ivan: implications for management. Biotropica. 2015;47(4):441-8. doi: 10.1111/btp.12231.

27. Raventós J, González E, Mújica E, Doak DF. Population viability analysis of the epiphytic ghost orchid (*Dendrophylax lindenii*) in Cuba. Biotropica. 2015;47(2):179-89. doi: 10.1111/btp.12202.

28. Robertson KM, Platt WJ. Effects of multiple disturbances (Fire and Hurricane) on epiphyte community dynamics in a subtropical forest, Florida, U.S.A. Biotropica. 2001;33(4):573-82. doi: 10.1111/j.1744-7429.2001.tb00216.x.

29. Rodríguez-Robles JA, Ackerman JD, Meléndez E. Host distribution and hurricane damage to an orchid population at Toro Negro Forest, Puerto Rico. Caribb. J. Sci. 1990;26(3-4):163-4.

30. Schnitzler A, Arnold C, Walter JM. Understanding the key mechanisms that govern climber/hemiepiphyte responses to a hurricane. J. Veg. Sci. 2015;26(4):778-90. doi: 10.1111/jvs.12279.

31. Tremblay RL. Ecological correlates and short-term effects of relocation of a rare epiphytic orchid after Hurricane Georges. Endanger. Species Res. 2008;5(1):83-90. doi: 10.3354/esr00114.

32. Vale Á, Rojas D, Álvarez JC, Navarro L. Distribution, habitat disturbance and pollination of the endangered orchid *Broughtonia cubensis* (Epidendrae: Laeliinae). Bot. J. Linn. Soc. 2013;172(3):345-57. doi: 10.1111/boj.12042.

33. Weaver PL. Impacts of Hurricane Hugo on the dwarf cloud forest of Puerto Rico's Luquillo Mountains. Caribb. J. Sci. 1999;35:101-11.

34. Wiegand T, Raventós J, Mújica E, González E, Bonet A. Spatio-temporal analysis of the effects of Hurricane Ivan on two contrasting epiphytic orchid species in Guanahacabibes, Cuba. Biotropica. 2013;45(4):441-9. doi: 10.1111/btp.12025.
